# Electro-photochemical Functionalization of C(sp^3^)–H bonds: Synthesis toward Sustainability

**DOI:** 10.1021/jacsau.4c00496

**Published:** 2024-09-09

**Authors:** Puja Singh, Burkhard König, Aslam C. Shaikh

**Affiliations:** † Department of Chemistry, Indian Institute of Technology Ropar, Rupnagar, Punjab-140001, India; ‡ Institute of Organic Chemistry, University of Regensburg, D-93040 Regensburg, Germany

**Keywords:** electrophotochemistry, C(sp^3^)−H bond activation, sustainability, enantioselective, late-stage functionalization

## Abstract

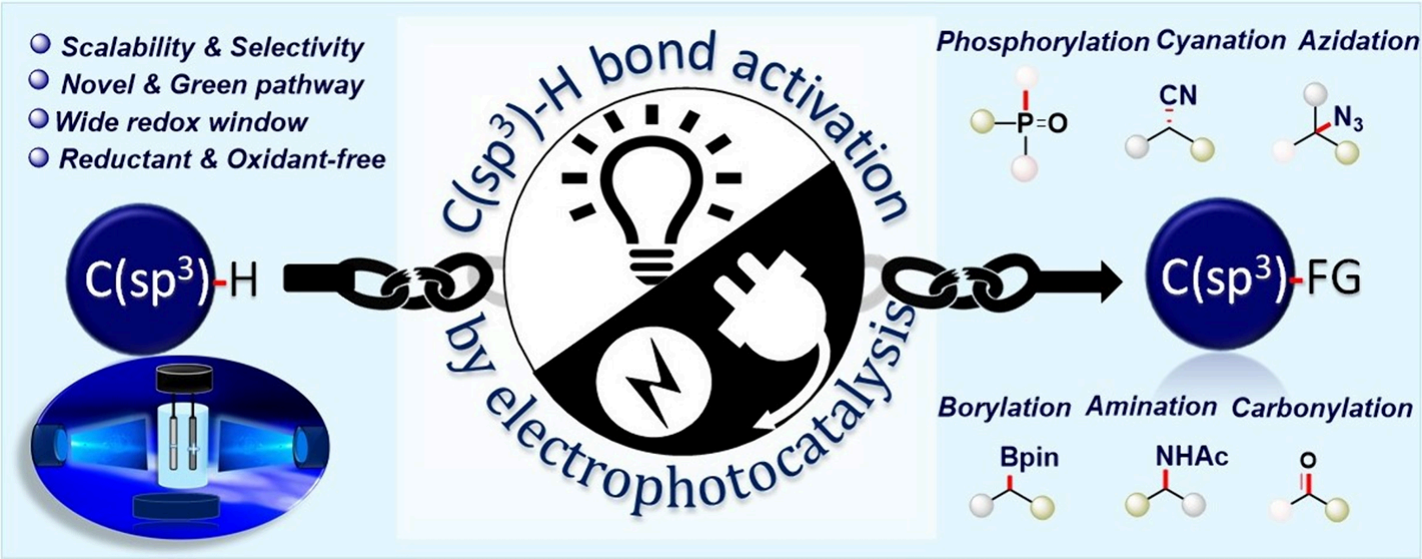

Over the past several decades, there has been a surge of interest in harnessing the functionalization of C(sp^3^)–H bonds due to their promising applications across various domains. Yet, traditional methodologies have heavily leaned on stoichiometric quantities of costly and often environmentally harmful metal oxidants, posing sustainability challenges for C–H activation chemistry at large. In stark contrast, the emergence of electro-photocatalytic-driven C(sp^3^)–H bond activation presents a transformative alternative. This approach offers a viable route for forging carbon–carbon and carbon–heteroatom bonds. It stands out by directly engaging inert C(sp^3^)–H bonds, prevalent in organic compounds, without the necessity for prefunctionalization or harsh reaction conditions. Such methodology simplifies the synthesis of intricate organic compounds and facilitates the creation of novel chemical architectures with remarkable efficiency and precision. This review aims to shed light on the notable strides achieved in recent years in the realm of C(sp^3^)–H bond functionalization through organic electro-photochemistry.

## Introduction

Activating C(sp^3^)–H bonds is one of the most efficient ways of synthesizing functionalized organic molecules from abundant and inexpensive feedstocks or in late-stage functionalization, e.g., for pharmaceuticals.^1−5^ Simple starting materials or fully functionalized molecules can be effectively activated and transformed, yielding target compounds. C(sp^3^)–H bonds are ubiquitous in all organic molecules, but their high redox potential (often greater than 3.0 V relative to SCE) and strong bond dissociation energy (BDE ∼ 96–101 kcal/mol) make the activation difficult.^6^ The small difference in reactivity between various C(sp^3^)–H bonds in molecules is a challenge in selectivity.^7−9^ Therefore, the search for a general and effective approach to C–H activation with high turnover and good selectivity that is industrially useful is continuing.

Transition metal catalysis,^10,11^ directing group-assisted or undirected methodology, and thermal-catalyzed cross-dehydrogenative coupling^12^ for C(sp^3^)–H bond activation have been explored in this context. Such strategies often require prefunctionalization, which produces waste. Employing coordination or chelation with transition metals, directing groups regulate the intrinsic steric/electronic characteristics and ultimately initiate site-specific C(sp^3^)–H bond activation. However, there are limitations. The addition and removal of a directing group often require harsh conditions and increase the number of reaction steps.^13^ Although these conventional approaches have significantly contributed toward the success of C(sp^3^)–H bond activation, there is room for improvement. Photochemical and electrochemical approaches may offer advantages in achieving C(sp^3^)–H functionalization.^14,15^ One of the main benefits of photoredox catalysis is the direct C(sp^3^)–H functionalization in inert compounds, which eliminates the need for directing groups and preactivation stages while also reducing the entire synthetic process in a mild environment. However, the activation of aliphatic C–H bonds still has limitations despite the great progress made. For instance, selectivity in C–H activation is still a daunting task in many cases.^16−18^ Nevertheless, this issue has been partially resolved by the photochemical hydrogen atom transfer (HAT) process or photoredox/metal dual catalysis.^19−21^ A broad range of photoinduced direct HAT catalysis processes are possible, such as formylation, alkylation, carboxylation, oxidation, alkynylation, vinylation, halogenation, and cyanation, which enable the selective introduction of functional groups in place of the original C–H bonds.^22−27^ However, the main obstacle to its widespread use is the small number of photocatalysts that can carry out direct HAT, which is confined to polyoxometallate, benzophenone, quinone, and uranyl cation families.^28,29^ Also, the regioselective activation of chemically similar C–H bonds remains a significant challenge. As a prime example, MacMillan first reported a photoredox-catalyzed Minisci-type C–H activation of aryl ethers which had a regioselective limitation for the nonsymmetric substrate.^30^ Another fundamental challenge of C–H activation, in terms of efficiency, is that it is typically an oxidation step. As every oxidation requires a stoichiometric terminal oxidation reagent, a coupled waste product is unavoidable. While this may be acceptable on a small scale or for the production of high-value compounds, it will preclude the use of the method on a larger scale. Additionally, a stoichiometric oxidation reagent is required to regenerate the photocatalyst. To overcome this limitation, electro-organic synthesis is an ideal approach to deal with waste generation by utilizing electrons as an oxidant and reductant.^31,32^ However, electro-organic synthesis, which involves activating C(sp^3^)–H bonds, has some limitations. These include issues with cross-dehydrogenative coupling,^33,34^ over-oxidation/reduction, a high ohmic drop between two electrodes, and ineffective mass transport at the electrode/bulk solution interface and electrode passivation.^35,36^ Therefore, it remains a demanding task to devise sustainable C(sp^3^)–H functionalization methods that are independent of the use of transition metals, expensive photocatalysts, and stoichiometric terminal oxidation reagents having minimal side reactions with considerable regio/enantioselectivity.

**Figure 1:**
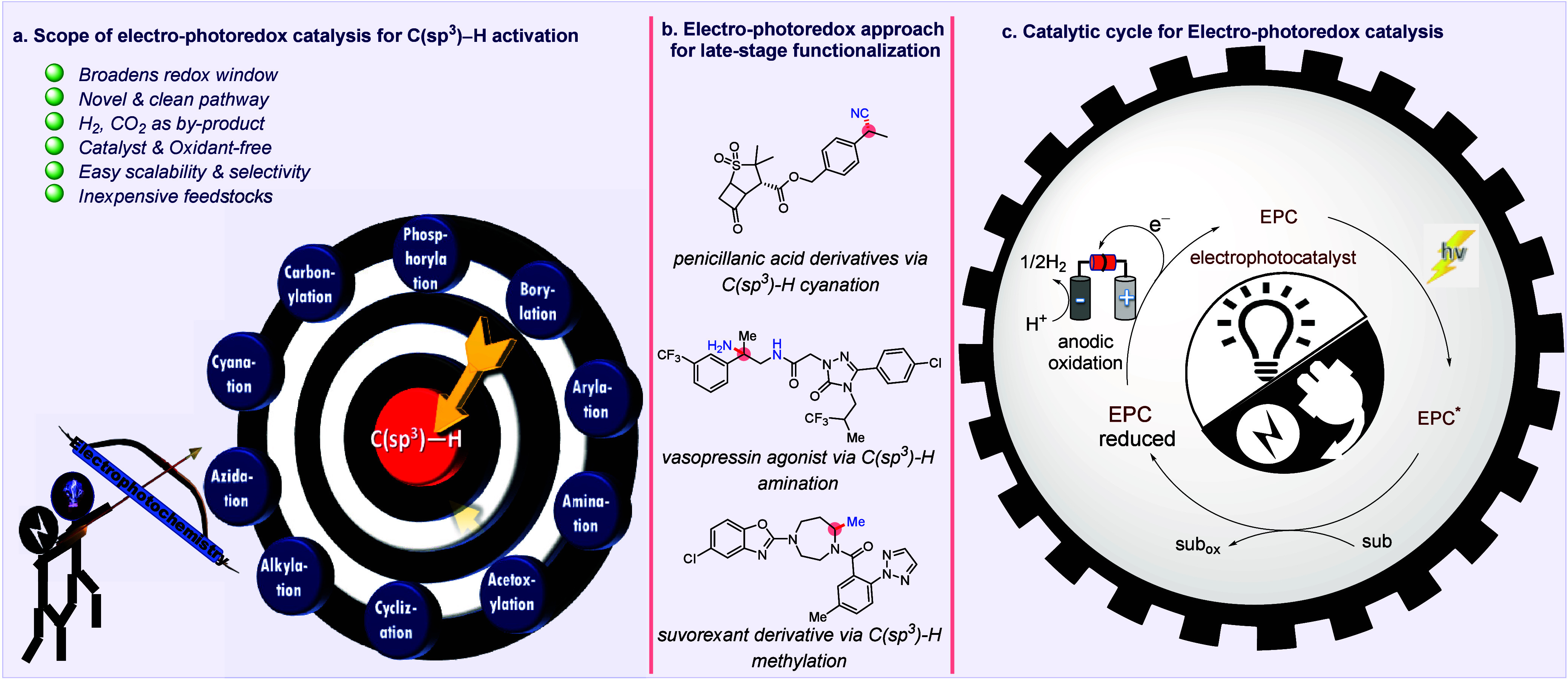
Electro-photoredox approach for targeting C(sp^3^)–H bond activation.

A possible remedy for these complications is provided by the combination of photoredox catalysis (PRC) with electrocatalysis (EC), i.e., electro-photo redox catalysis (EPRC).^37−39^ Electrophotochemistry combines the merits of both electrochemistry and photocatalysis, while overcoming their shortcomings. Recent breakthroughs in the field of electro-photocatalysis highlight that a plethora of challenging and important reactions, such as C(sp^3^)–H functionalization, could be explored by employing this technique.^40−42^ The integration of electrochemistry with photochemistry is anticipated to improve the selectivity and functional group tolerance under mild conditions, promote atom economy, and expand the redox window of SET processes in a single catalytic cycle (Figure 1c). In addition, this method allows for the activation of an inert substrate (such as a C(sp^3^)–H bond) with a high oxidative/reductive potential at a considerably lower redox potential. Coupling of PRC with EC allows the oxidative regeneration of the photoredox catalyst by electricity at the anode and couples this with dihydrogen generation (the terminal oxidants are protons) at the cathode. EPRC could eliminate the addition of external oxidants by the proper selection of the cell potential. There have been numerous reviews and articles on the subject of electrophotoredox catalysis in general. This review is intended to provide insights into the electro-photocatalytic functionalization of C(sp^3^)–H bonds with some selected examples, possible mechanisms, and late-stage functionalizations of natural products, pharmaceuticals, and agrochemicals to date. To make it more accessible, we have divided this review into five parts based on the transformation of C(sp^3^)–H bonds into C(sp^3^)–X bonds, where X can be C, N, O, B, P, S, etc. We conclude by discussing future directions for research on C–H functionalization, with a focus on the obstacles that need to be removed for this kind of methodology to be better used in both academia and industry.

## 2 Electro-photochemical C(sp^3^)–H Functionalization

### 2.1 Functionalization of C(sp^3^)–H Bond into C(sp^3^)–C Bond

**Scheme 1:**
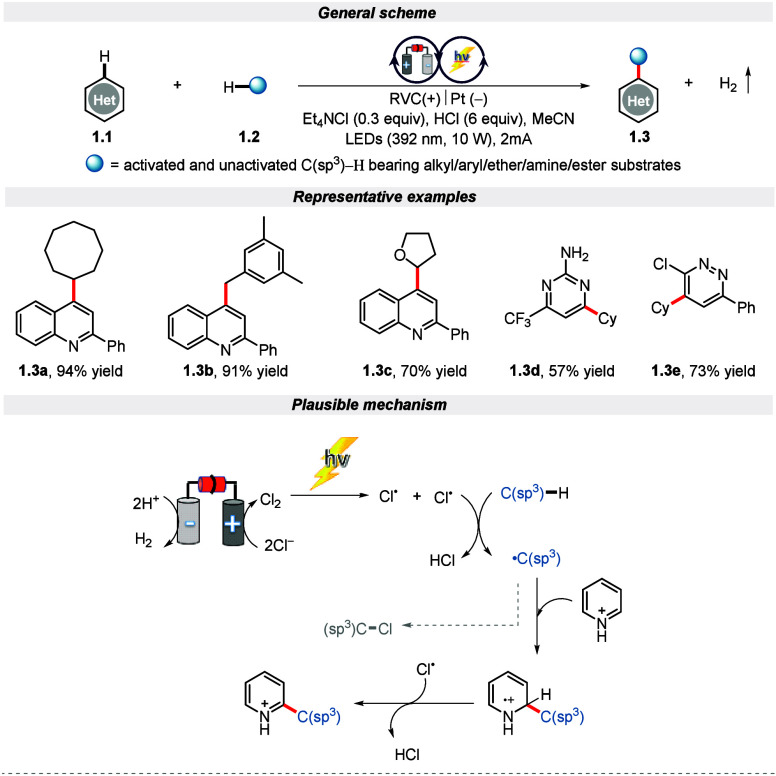
Electro-photochemical Dehydrogenative Cross-Coupling of Heteroarene with C(sp^3^)–H Bond-Containing Substrates

The construction of the C–C bond is one of the most explored organic transformations in synthesis since it is the basis of organic molecules.^43^ Extensive research has been conducted in photoredox catalysis and electrochemistry to form the C–C bond via C(sp^3^)–H bond activation. However, a combination of both fields provides an alternative and potentially sustainable approach to construct C–C bonds through direct activation of C(sp^3^)–H bonds.^44^ In light of this, Xu and co-workers in 2020 explored a dehydrogenative cross-coupling strategy that enables the formation of C–C bonds by incorporating the EPRC methodology (Scheme 1).^45^ This innovative reaction scheme offers an exciting new approach that utilizes metal-free and oxidant-free conditions for coupling diverse heteroarenes with activated and unactivated C(sp^3^)–H donors. Electron-deficient heteroarenes, such as quinoline, pyrimidines, isoquinolines, quinoxaline, and pyridines, are compatible for alkylation reaction with C(sp^3^)–H bond bearing cyclic alkanes, ester, amines, amides, and ethers. Clear evidence obtained by the control experiments outlines that light and electricity are essential for the formation of an alkyl radical intermediate for a successful reaction. Further, the mechanistic cycle suggests that the chlorine radical formed at the anode via homolytic cleavage of chlorine molecules on subsequent irradiation converts the C(sp^3^)–H bond to a carbon-centered alkyl radical intermediate. Eventually, this intermediate attacks the heteroatom to furnish the corresponding product (Scheme 1). Remarkably, the authors showed the synthetic utility of this protocol by performing a reaction at a gram or decagram scale with a minimal yield drop. Very recently, Wang and co-workers adopted a similar protocol for electro-photocatalytic cross-coupling of heteroarenes with unactivated C(sp^3^)–H compounds by utilizing 9,10-phenanthrenequinone as a photocatalyst.^46^

**Scheme 2:**
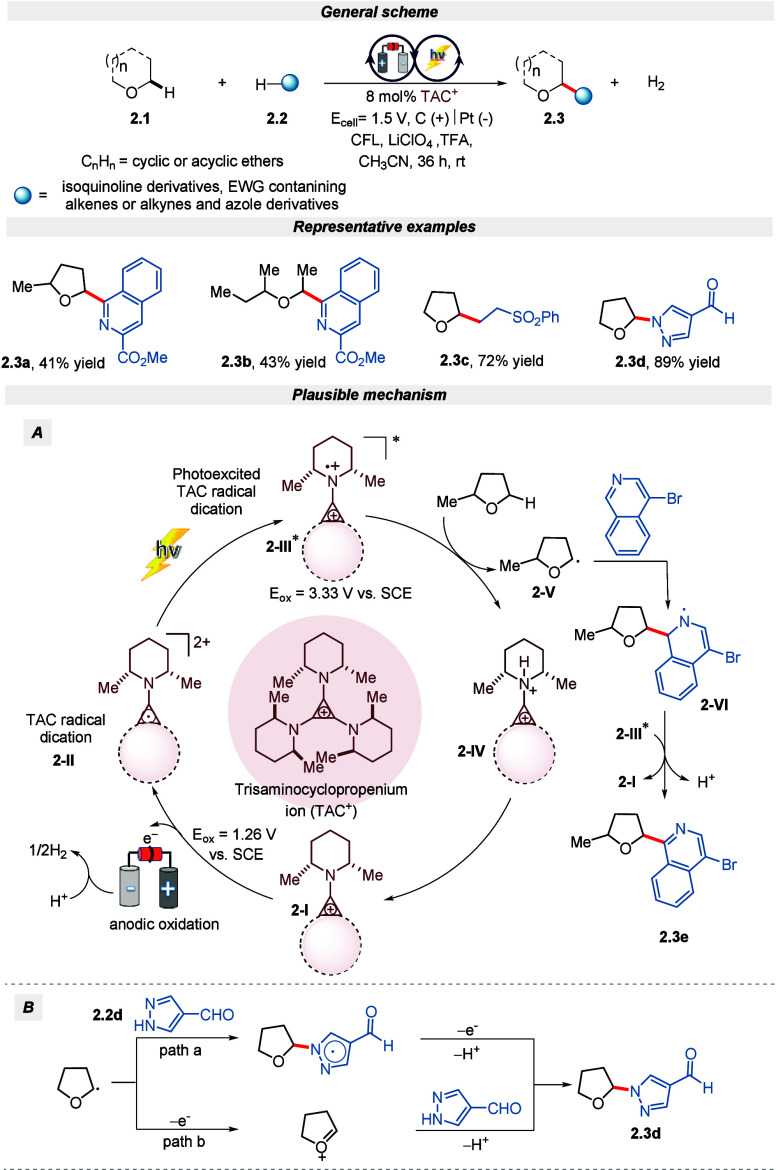
Electro-photochemical C(sp^3^)–H Functionalization of Ethers

Tandem electro- and photoredox catalysis is known to generate superoxidants and reductant species for the activation of the inert substrate.^47,48^ With the same intention, Lambert and colleagues demonstrated the use of a trisaminocyclopropenium ion (TAC) as an electro-photocatalyst under visible light for the C(sp^3^)–H functionalization of ethers with good regioselectivity and scalability (Scheme 2).^49^ The efficacy of this protocol in the conversion of cyclic or acyclic ethers and heteroarenes is noteworthy, making it a promising method for various applications. According to the author, the anodic oxidation of TAC (with *E*_ox_ = +1.26 V vs SCE) produces a photoactive radical dication (**2-II**). This species absorbs visible light and transforms into the superoxidant intermediate (**2-III**, with *E*_red_ = +3.33 V vs SCE), having an aminyl radical cation character. As shown in Scheme 2, **2-III** bears sufficient strength for an effective substrate oxidation process. Further, transferring a hydrogen atom from the ether substrate to intermediate radical cation **2-IV** leads to the production of the corresponding substrate radical **2-V**. Upon interaction with isoquinoline followed by a second oxidation along with simultaneous deprotonation, the desired product is obtained. Hence, it was acknowledged that C–C coupling products proceed via an intermediate, whereas coupling with azole followed two pathways, as depicted in Scheme 2.

**Scheme 3:**
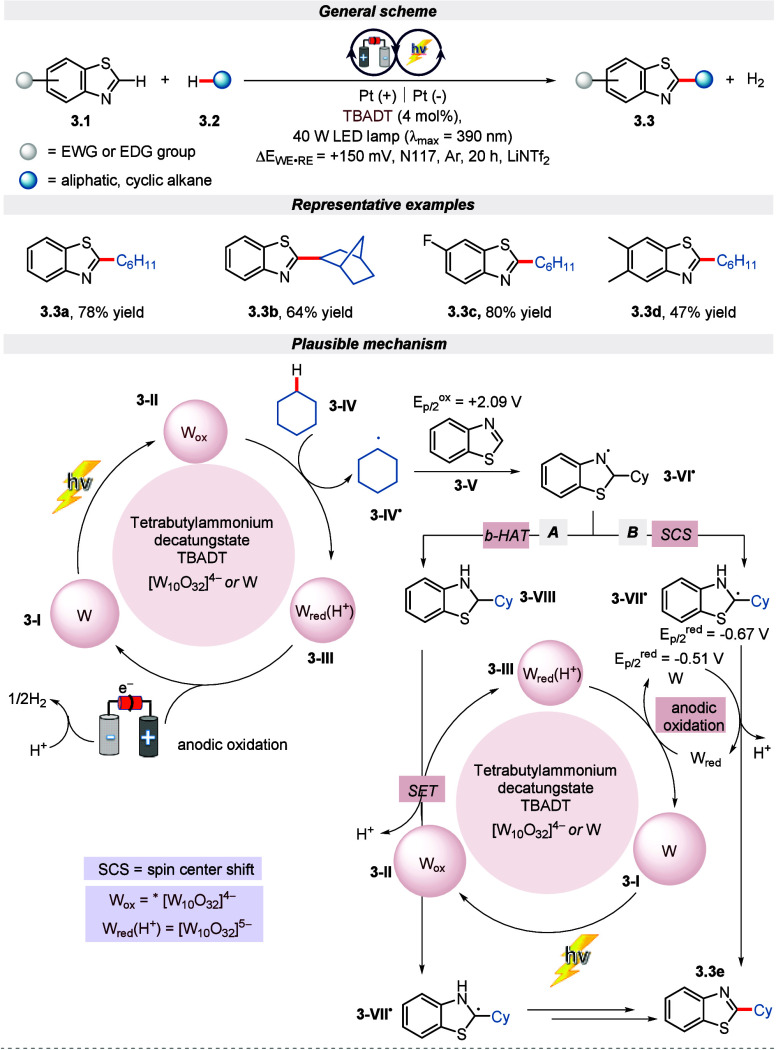
Electro-photochemical Dehydrogenative Cross-Coupling of Benzothiazole with C(sp^3^)–H Bonds

Later in 2021, Ravelli and co-workers reported an electro-photochemical activation of the C(sp^3^)–H bond using tetrabutylammonium decatungstate (TBADT) as an electro-photocatalyst.^50^ It is worth highlighting that the excited state of TBADT is responsible for activating the C–H bond via the HAT step^51^ in the presence of aliphatic hydrogen donor alkanes. Control experiments reveal that if the reaction is shielded from either light, photocatalyst, or electricity, then the reaction does not proceed. Both electron-rich and electron-deficient benzothiazole analogues underwent dehydrogenative coupling with cyclic and acyclic alkanes, generating excellent yields of the desired product. The mechanism for the C–C coupling reaction is initiated by the excited state of TBADT **3-II** after light irradiation. Compound **3-II** converts the substrate **3-IV** to radical **3-IV**^**•**^, which adds to the C2 position of benzothiazole and forms radical adduct **3-VI**^**•**^. Further, as depicted in Scheme 3, **3-VI**^**•**^ reacts via either a back-HAT pathway or a spin center shift pathway to furnish the desired product.

**Scheme 4:**
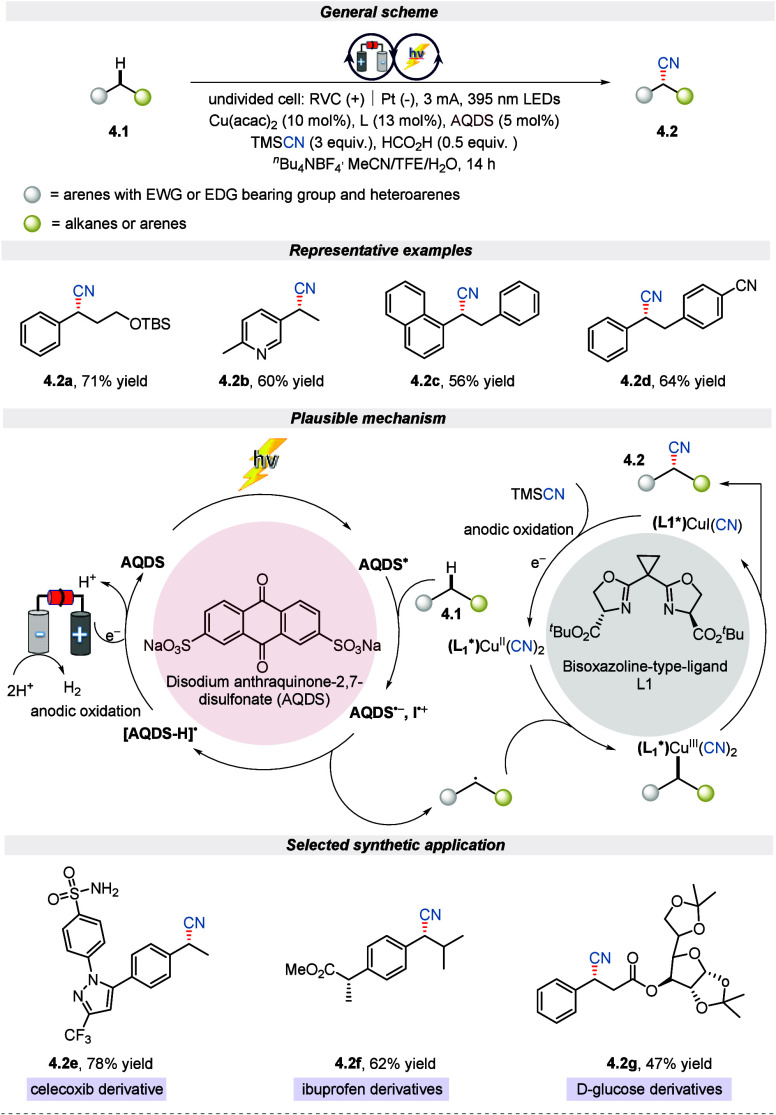
Electro-photochemical Enantioselective Cyanation of Benzylic C(sp^3^)–H Bonds

Enantioselective functionalization of C(sp^3^)–H bonds is an attractive approach to access chiral complexes.^52,53^ However, achieving it without transition metal catalysis, without a directing group, and in the presence of a radial-based approach with great selectivity is always challenging.^54,55^ By employing an electro-photochemical strategy, Xu et al. in 2022 disclosed enantioselective cyanation of benzylic C(sp^3^)–H bonds with excellent site selectivity.^56^ The reaction conditions exhibited a wide substrate scope with late-stage functionalization of complex bioactive molecules. As conveyed in Scheme 4, this electron-transfer-based mechanism using disodium anthraquinone-2,7-disulfonate (AQDS) as an electro-photocatalyst and copper as a cocatalyst consists of two catalytic cycles. The first catalytic cycle begins with the electron transfer of excited AQDS with alkylarene **4.1** to form an ion-radical pair (AQDS^**•–**^).^57^ Following a proton transfer, these ionic radical species yield a semiquinone radical (AQDS–H^**•**^) and a benzylic radical that further reacts with copper complex (L1)Cu^II^(CN)_2_ to generate Cu(III) species. This Cu(III) species experiences reductive elimination to deliver the product (III). Pharmaceutical drugs like celecoxib, ibuprofen, and d-glucose derivatives were cyanated with good yield. Additionally, the enantioselective cyanation reaction was smoothly executed on a gram scale without any loss of efficiency or productivity.

**Scheme 5:**
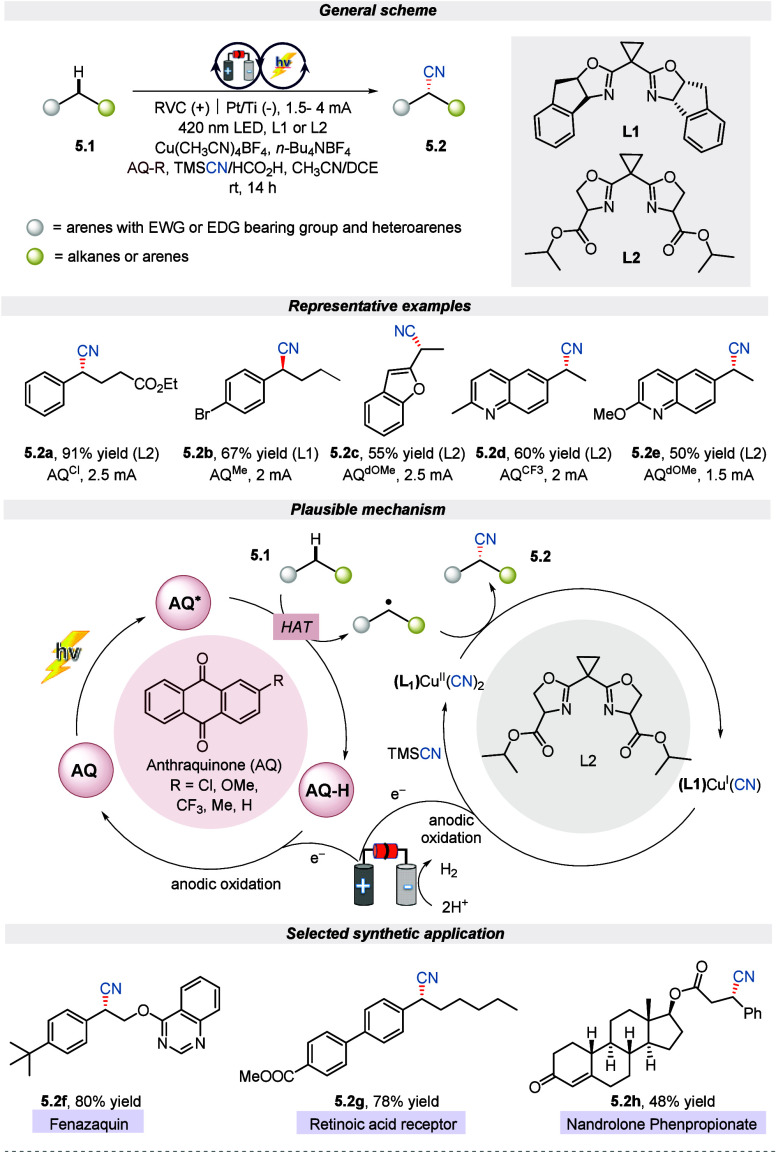
Electro-photochemical Enantio-Selective Cyanation of Benzylic C(sp^3^)–H Bonds

Adopting a similar strategy Liu et al. portrayed another enantioselective cyanation of the benzylic C(sp^3^)–H bond by harnessing dual metallo-electro-photoredox catalysis.^58^ This decoupled radical relay strategy exhibited an extensive substrate scope along with late-stage diversification of bioactive molecules and natural products. The tunable electronic properties of the photocatalyst anthraquinone along with a ligand (L1 or L2) and the applied voltage make this approach commendable and highly enantioselective in good to excellent yield. Most importantly, this strategy avoids the use of external oxidants such as N-fluoro-succinimide (NFSI), as used in the previous case for the successful cyanation of benzylic C(sp^3^)–H bonds. The mechanistic cycle depicted in Scheme 5 suggested that the photoexcited anthraquinone (AQ) acts as a HAT acceptor^59^ and converts the alkylarene to a benzylic radical intermediate along with reduced AQ-H. Following this, the benzylic radical intermediate is grasped by the LCu^(II)^(CN)_2_ complex which eventually delivers the product. Finally, a terminal oxidant is utilized to reoxidize the reduced AQ-H and LCu^(I)^(CN) to close the catalytic cycle at the anode.

**Scheme 6:**
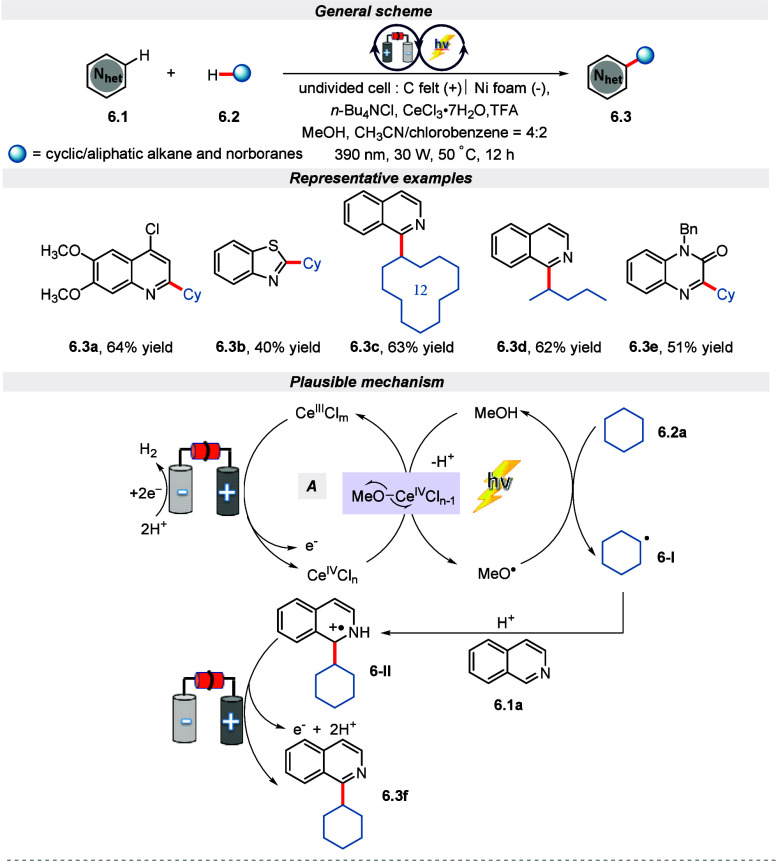
Electro-photochemical Alkylation of *N*-Heteroarene Using Alkanes via C(sp^3^)–H Bond Activation

In 2022, Zeng et. al. reported a cerium-catalyzed electro-photocatalytic reaction design for the alkylation of *N*-heteroarenes with unactivated alkanes via C(sp^3^)–H bond activation.^60^ Satisfyingly, this strategy worked for an extensive range of unactivated alkanes, which includes cyclic and aliphatic alkanes, along with norborane systems. Further, the substrate scope was also compatible with *N*-heteroarenes namely isoquinolines, quinolines, quinoxaline, phenanthridine, pyridine, and benzothiazole with substituted electron-rich/deficient functional groups. A plausible reaction pathway is shown in Scheme 6; the reaction begins with the oxidation of Ce(III) to Ce(IV) in the presence of *n*-Bu_4_NCl. This is followed by light-mediated ligand-to-metal charge transfer (LMCT) in the presence of MeOH, resulting in the formation of the MeO^**•**^ radical. Thereafter, this electrophilic MeO^**•**^ abstracts hydrogen from cyclohexane via the HAT event to remit carbon-centered radical **6-I**. Ultimately, radical **6-I** adds to the heteroarene-substrate **6-II** which upon further oxidation, followed by loss of protons, leads to the alkylated product.

**Scheme 7:**
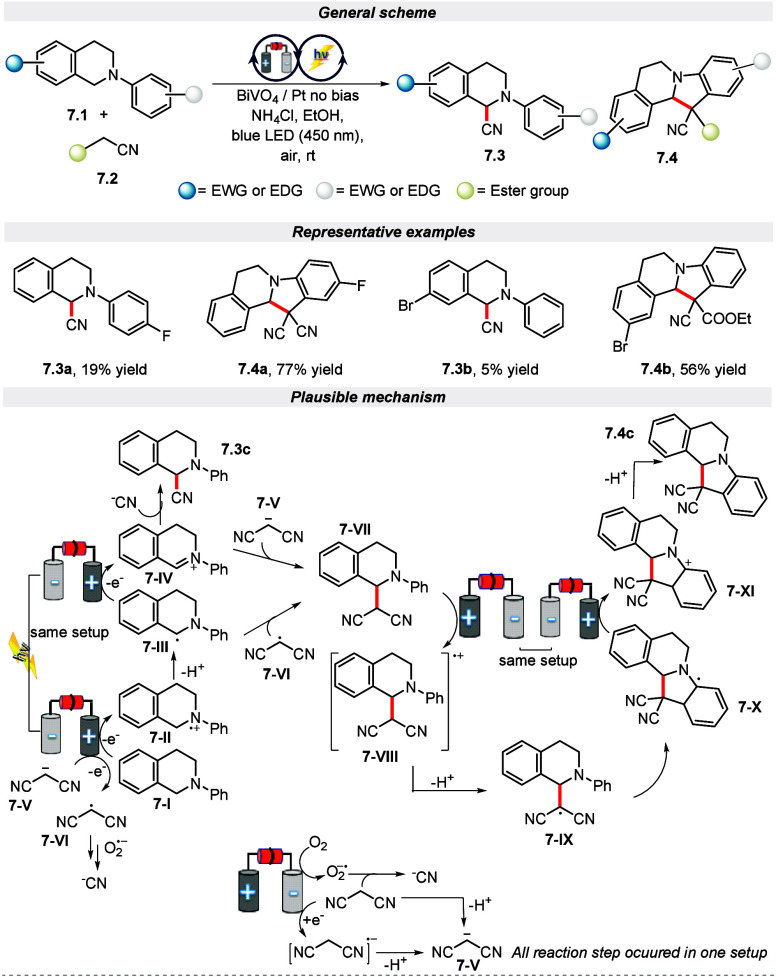
Electro-photochemical Synthesis of *N*-Bearing Fused Ring via C(sp^3^)–H Bond Activation

Similarly, in the same year, Wu et al. developed a novel electrophotocatalytic protocol to synthesize *N*-bearing fused ring via the C(sp^3^)–H bond activation of tetrahydroisoquinoline derivatives utilizing an m-BiVO_4_ film as a photoanode in blue LED (Scheme 7).^61^ Gratifyingly, this strategy was compatible with *para*-substituted N-aryltetrahydroisoquinolines bearing both electron-donating and weak inductively electron-withdrawing groups to furnish the cyclized product **7.4** in good yield but yielded a small amount of the cyanated product **7.3**. Whereas, in the case of strong electron-withdrawing groups, the yield and the selectivity for the products **7.4** and **7.3** got reversed. Similarly, in the case of *meta*-substituted *N*-aryltetrahydroisoquinolines bearing a strong electron-withdrawing group and strong steric hindrance, furnished the cynated product **7.3** in good yield. The proposed mechanism for this protocol initially began with the generation of electron–hole pairs by the excited BiVO_4_ photoelectrode under visible light irradiation. Further, the holes generated on the surface of photoanode oxidizes **7-I** to **7-II** which on deprotonation generates the radical **7-III**. The so-formed radical **7-III** is further oxidized to iminium ion intermediate **7-IV**, which, upon nucleophilic attack of the CN^–^ anion, yields the product **7.3c**. The formation of anion CN^–^, **7-V**, and radical **7-VI** is achieved through oxidation or reduction. Subsequently, intermediate **7-VII** is achieved through either the radical coupling of **7-VI** and **7-III** or by nucleophile attack of **7-V** on **7-IV**. Further, the oxidation of **7-VII** to give **7-VIII** is achieved on the photoanode which upon deprotonation gives the radical **7-IX**. Finally, the desired product is achieved via the addition of a benzene ring and an oxidation/deprotonation process.

**Scheme 8:**
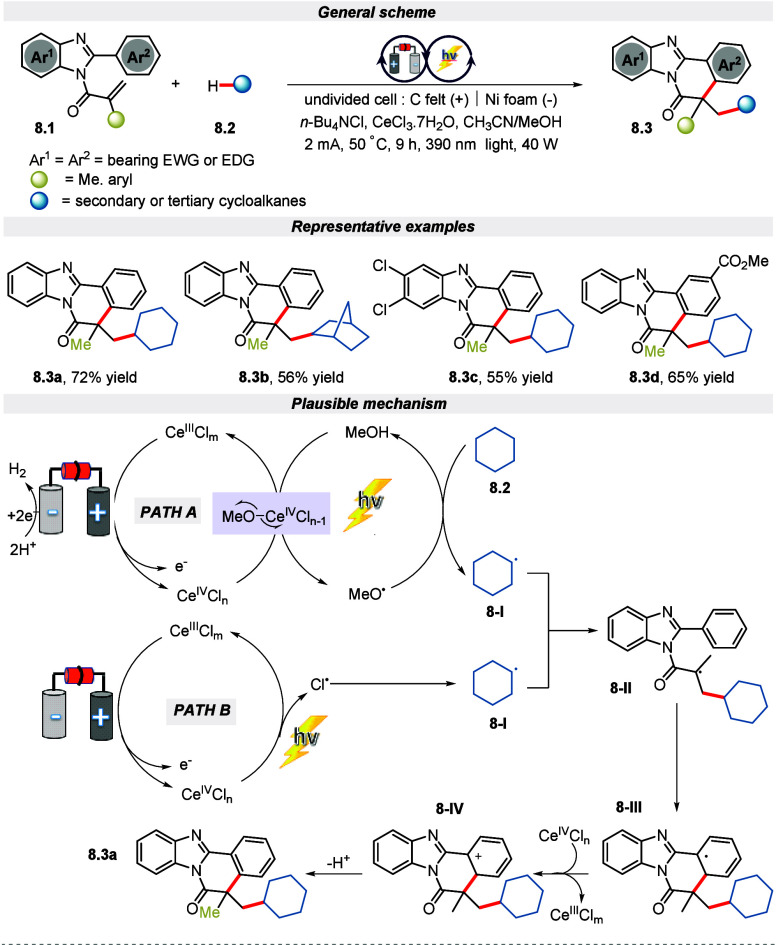
Electro-photochemical Synthesis of Benzimidazole-Fused Isoquinolines via C(sp^3^)–H Bond Activation of Alkanes

In a recent publication from Zeng and his co-workers, they elegantly demonstrated a cerium-catalyzed electro-photocatalytic route for the synthesis of benzimidazole-fuse isoquinolines and other *N*-bearing polycyclic compounds via the activation of C(sp^3^)–H bond.^62^ It is worth pointing out that this oxidant-free approach for radical addition/cyclization of unactivated alkane features a high atom economy. Both electron-donating and -withdrawing benzimidazole fused quinoline along with secondary or tertiary alkanes were competent with this reaction condition. According to the mechanistic studies along with the previous reports,^63,64^ a mechanism is designed that reveals the formation of the complex MeO–Ce^IV^Cl_*n*–1_ by the anodic oxidation of Ce^III^. This complex then underwent homolysis to afford the MeO^**•**^. Meanwhile, via HAT from cyclohexane, the MeO^**•**^ changes to MeOH and leads to a cyclohexyl radical as well. Finally, the cyclohexyl radical undergoes addition or cyclization with the substrate to deliver intermediate **8-III**. This intermediate subsequently encounters single electron transfer (SET) oxidation by Ce(IV) followed by deprotonation to furnish the cyclized product (Scheme 8).

**Scheme 9:**
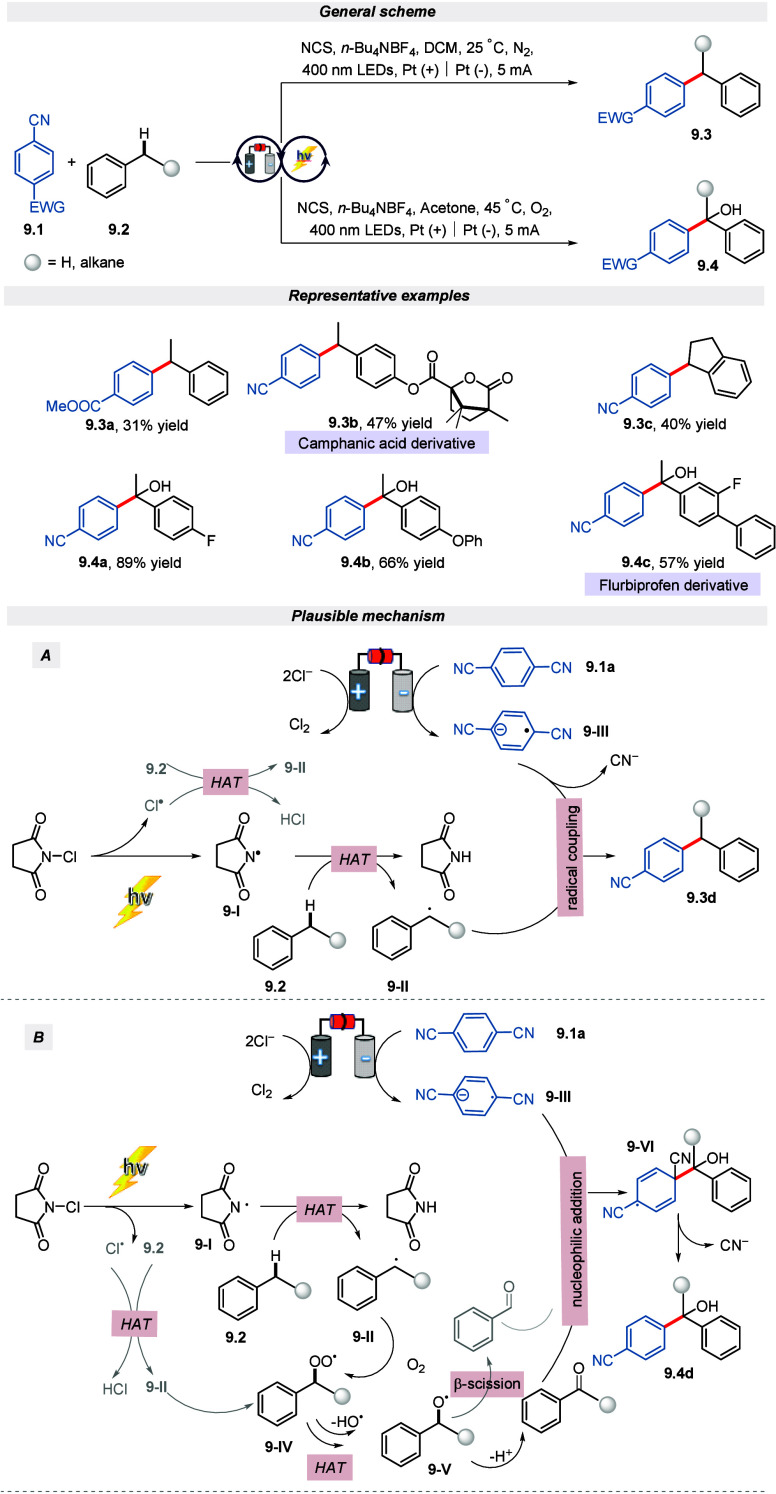
Electro-photochemical Arylation of Benzylic C(sp^3^)–H Bonds

In the same year, Wang and his group devised an electro-photocatalytic benzylic C(sp^3^)–H arylation reaction for the construction of diaryl alcohols and diaryl alkanes with exceptional selectivity, wide scope and scalability.^65^ On the grounds of previous literature^66^ and control experiments, the following mechanism was proposed for direct and oxygenative arylation (Scheme 9). To initiate the direct arylation reaction, the photoexcited *N*-chloro succinimide (NCS) changes to succinimide radical **9-I**, which subsequently extracts a hydrogen atom from alkylbenzene to give benzyl radical **9-II**. Thereafter, **9-II** reacts with a cathodically formed radical anion **9-II**I to furnish the product. On the other hand, on the appearance of oxygen, the benzyl radical **9-II** forms a superoxide species **9-IV** which subsequently, via HAT event and homolysis delivers the alkoxy radical **9-II**. Next, an alkyl radical and the equivalent aldehyde are produced via the β-scission of **9-II**. Further, species **9-III** upon nucleophilic addition to the corresponding aldehyde/ketone gives the intermediate **9-VI** which on subsequent reduction and elimination of cyanide yields the desired product.

**Scheme 10:**
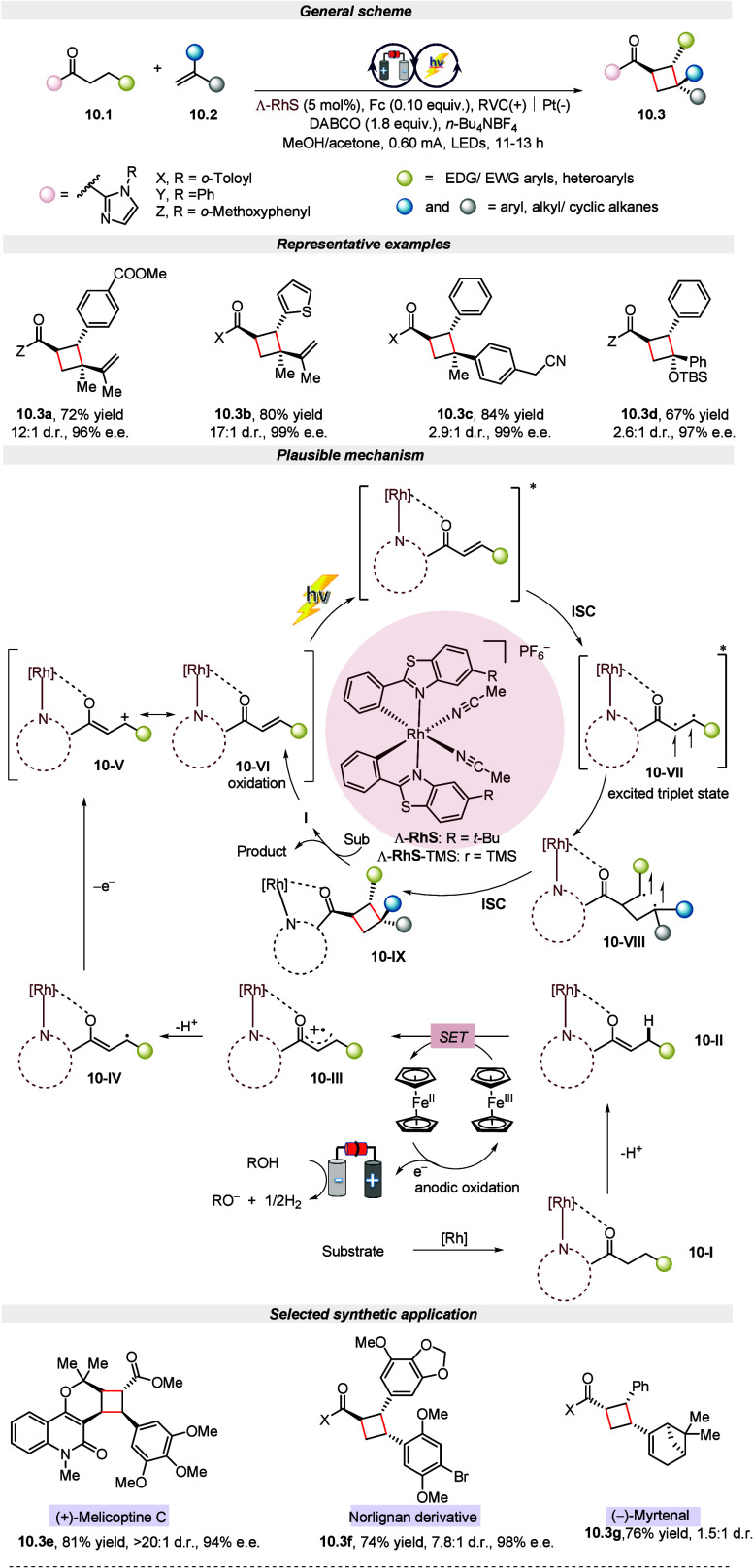
Electro-photochemical [2 + 2] Cycloaddition via Activation of Two C(sp^3^)–H Bonds

In 2023, an enantioselective and diastereoselective synthesis of chiral cyclobutane via dehydrogenative [2 + 2] cycloaddition by taking advantage of electro-photocatalytic chemistry was reported by Meggers and colleagues.^67^ Notably, this approach simultaneously activates the two C(sp^3^)–H bonds to convert into consecutive carbon stereocenters in an undivided cell equipped with reticulated vitreous carbon (RVC) as an anode and a platinum-plate cathode under blue light irradiation. The authors underlined that this one-pot reaction was tolerant to a wide range of electron-rich and electron-deficient ketones with alkenes such as styrenes, enyne, vinyl ether, and all other internal alkenes. Moreover, the protocol was also amenable to gram-scale reaction along with late-stage functionalization for the synthesis of the natural products melicoptine C, norlignane, myrtenal, and many others. The synthetic route to create the desired product starts by combining rhodium catalysts with alkyl ketones to form a bidentate-coordinated complex **10-I**. This **10-I** undergoes deprotonation to form enolate **10-II**, which, on oxidation by SET using oxidized ferrocene (Fc^+^) at the anode, forms **10-III**. Next, intermediate **10-III**, under acidic conditions, loses a proton and electron to form **10-V**, which is a tautomer of **10-VI**. After photoexcitation and internal system crossing (ISC), enone intermediate **10-VI** transforms into triplet state intermediate **10-VII**. Upon the addition of an alkene to **10-VII**, a triplet diradical intermediate **10-VIII** is generated. Ultimately, **10-VIII** undergoes ISC, resulting in the production of a Rh-coordinated cyclobutane and the subsequent release of the desired product (Scheme 10).

**Scheme 11:**
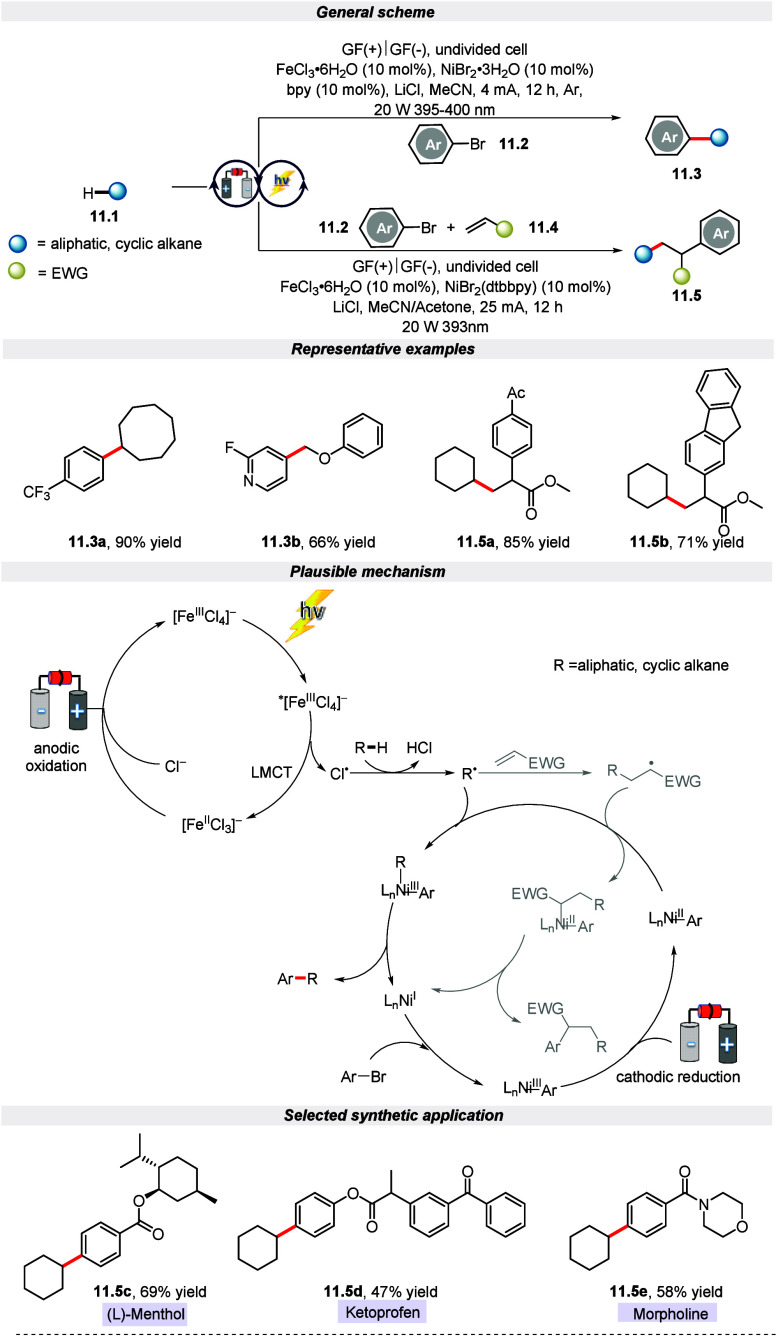
Electro-photochemical C(sp^3^)–H Bonds Alkylation and Arylation of Alkanes

In the recent publication, Lu and his group reported an electrophotocatalytic strategy for two-component C(sp^3^)–H arylation and three-component C(sp^3^)–H alkylation of alkanes via paired oxidative and reductive catalysis.^68^ It is worth highlighting that the selectivity of the reaction between C(sp^3^)–H arylation and C(sp^3^)–H alkylation can be fine-tuned by controlling the light source and the applied current. Further, the protocol demonstrated broad utility and functional-group tolerance with over 70 examples. The mechanistic investigations suggested that the electrophotocatalytic approach involves the transformation of C(sp^3^)–H into carbon radicals. This carbon radical is formed by chlorine radicals, which are generated through the light-induced LMCT of [FeCl_4_]^−^. Simultaneously, aryl bromide’s oxidative addition leads to Ni(III) species formation, which further gets reduced at the cathode to form the Ni(II) complex. Finally, the so-formed aryl radical combines with the Ni(II) complex, leading to Ni(III)(aryl)(alkyl) species, which, on reductive elimination, furnishes the required product. Notably, multicomponent C(sp^3^)–H alkylation reactions can be formed if an alkene is employed as a linkage in this process. Additionally, the method’s compatibility was illustrated at a preparatory level and utilized in the late-stage diversification of pharmaceutical derivatives and natural products (Scheme 11).

**Scheme 12:**
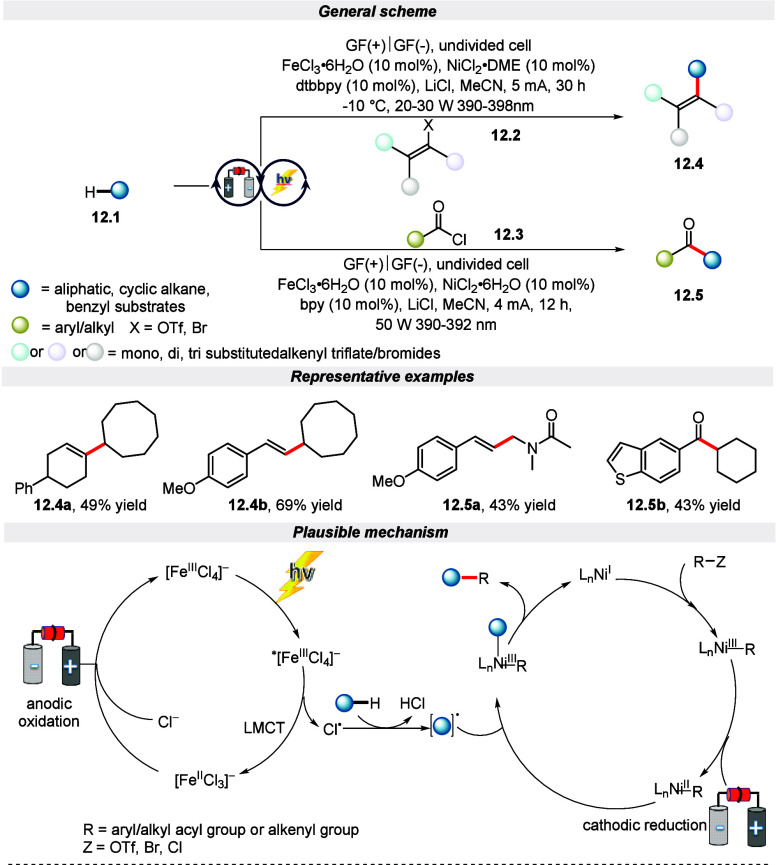
Electro-photochemical C(sp^3^)–H Bond Alkenylation

In the same year, a comparable paired electrolysis approach was developed by Lu and colleagues for the functionalization of alkanes through the activation of C(sp^3^)–H bonds (Scheme 12).^69^ This method utilized iron and nickel catalysts as electron shuttles to activate anodic and cathodic reactants simultaneously in a single cell, thus avoiding interference. It is noteworthy to mention that C(sp^3^)–H alkenylation and acylation of alkanes with ultralow oxidation potential are achieved using this protocol to construct mono- or multisubstituted olefins and ketones under mild conditions. Further, the author highlighted the generality of this protocol with a broad range of structurally diverse mono-, di-, and trisubstituted alkenyl triflate/bromides with good to excellent yield. Additionally, this strategy follows a similar kind of mechanistic route, as showcased and discussed in Scheme 11.

**Scheme 13:**
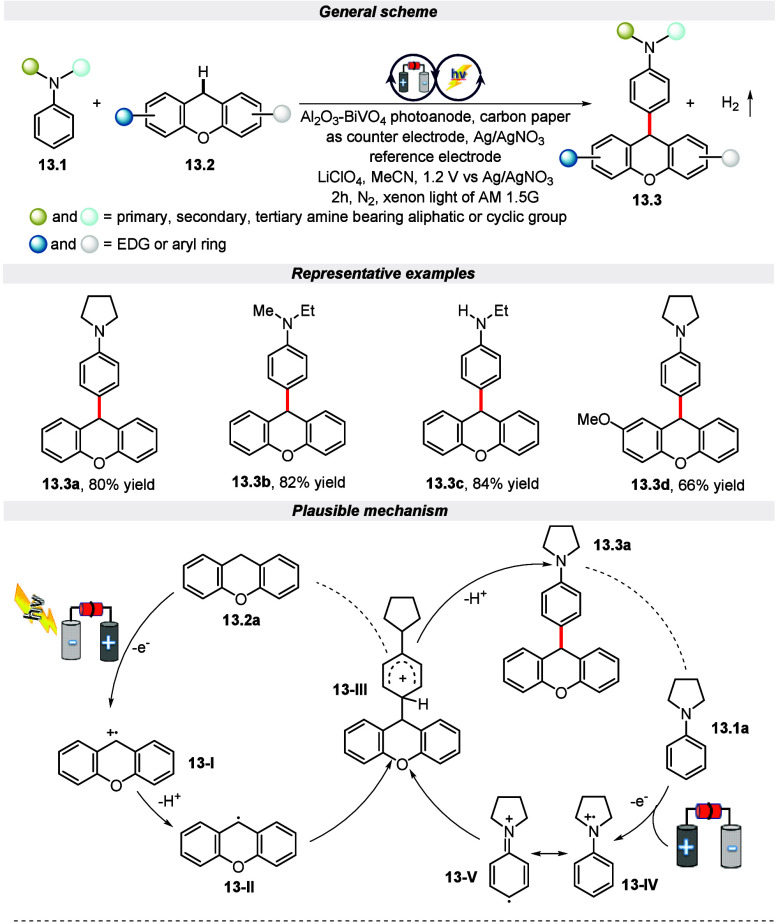
Electro-photochemical C–H Bond Activation of Amines and Xanthenes to Form Subsequent C–C Coupled Compounds

In the year 2023, Tang and his co-workers reported C–H bond activation of amines and xanthenes to form subsequent C–C coupled compounds by employing Al_2_O_3_–BiVO_4_ as a photoanode via the electrophotocatalysis approach (Scheme 13).^70^ It is worth mentioning that this method exhibited excellent tolerance toward active primary, secondary, and tertiary amines bearing electron-rich and electron-deficient groups. Additionally, xanthenes bearing 2-methyl, 2-meth oxyl, 1,3-dimethyl, and 4-methoxyl groups on the aryl rings proceeded smoothly to furnish the desired products in acceptable yield. Further, as depicted in the Scheme 13, the mechanistic cycle begins with the generation of electron–hole pairs by visible light excited photoanode. Subsequently, the hole migrates to the Al_2_O_3_–BiVO_4_ surface to oxidize **13.1a** to form its radical cation **13-IV**. Simultaneously, xanthene **13.2a** is also oxidized to **13-I**, which, upon deprotonation, forms a **13-II** radical intermediate. Following this, intermediate **13-III** is formed by the coupling of **13-II** and **13-V** which underwent deprotonation to furnish desired product **13.3a**.

### 2.2 Functionalization of C(sp^3^)–H Bond into C(sp^3^)–O Bond

**Scheme 14:**
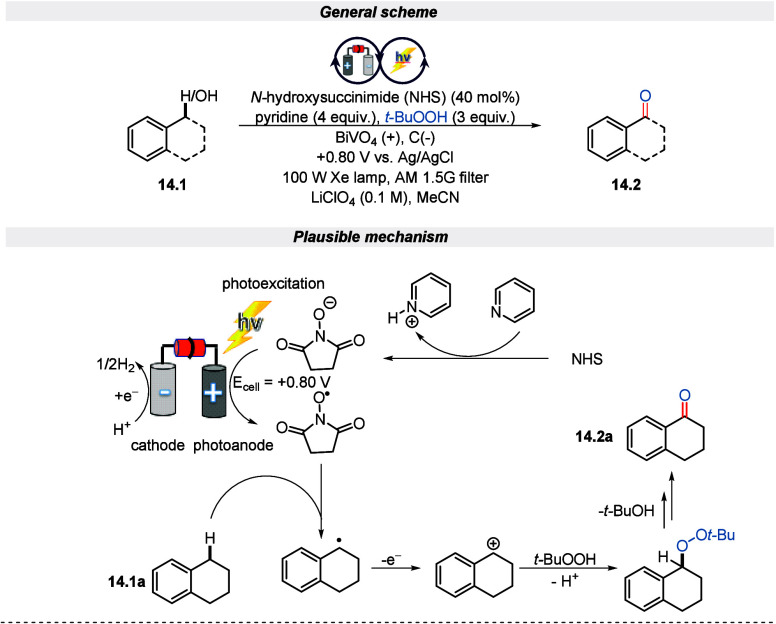
Electro-photochemical Oxidation of C(sp^3^)–H Bonds into Carbonyl Functionality

The thermal, electrochemical, or photochemical oxygenation of C–H bonds^71,72^ is a straightforward tool to install oxygen functionalities into organic molecules which has found application in drug development.^73^ However, this type of strategy is challenging due to the risk of overoxidation, site selectivity, and undesired side products. Also, oxygenating multiple C–H bonds are demanding. Ergo, innumerable scientific communities are trying to develop sustainable strategies that could overcome the aforementioned challenges. Considering this issue, Berlinguette and associates disclosed an *N*-hydroxysuccinimide (NHS) mediated C(sp^3^)–H oxidation of benzyl alcohol, tetralin, and cyclohexene under an electro-photochemical setup by using BiVO_4_ as a photoanode.^74^ It was observed that compared to classical electrochemistry, the oxidation of organic substrates was achieved with a 60% reduction in electrical energy. Although the yield of the product was low, it is worth noting that the solar-to-electricity efficiency (η = 1.3%) is comparable to that of conventional photoelectrochemical water oxidation (η = 1.7%). This is due to the higher value of the organic compounds produced, and it represents a promising step toward more efficient and sustainable chemical processes. Hence, a minimum of 1 day of continuous photochemical conversion is made possible by the significantly improved semiconductor photostability that results from using organic media instead of water (Scheme 14).

**Scheme 15:**
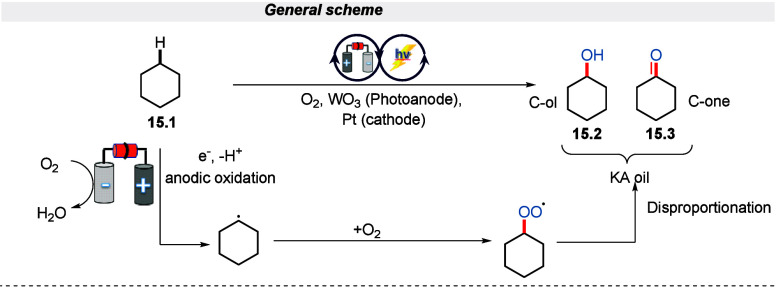
Electro-photochemical Oxidation of Unactivated Alkanes C(sp^3^)–H Bonds

In 2018, Sayama and his colleagues utilized an electro-photochemical method to accomplish the oxidation of unactivated alkanes to alcohols in aerobic conditions with WO_3_ as a photoanode under the irradiation of 365 nm wavelength of light.^75^ The reaction for the synthesis of cyclohexanol (the so-called KA oil) was achieved with a high partial oxidation selectivity and a high current utilization ratio. This unprecedented method proceeds via the single-electron transfer process for the production of cyclohexyl radical which further reacts with oxygen to deliver a cyclohexylperoxyl radical. This cyclohexylperoxyl radical then disproportionates to form the corresponding alcohol and ketone, as showcased in Scheme 15.

Similarly, in the year 2021, Xiong et al. demonstrated an electrophotochemical strategy to convert methane to ethylene glycol by employing WO_3_ photoanode with ethylene glycol production rate of 0.47 μmol cm^–2^ h^–1^.^76^

**Scheme 16:**
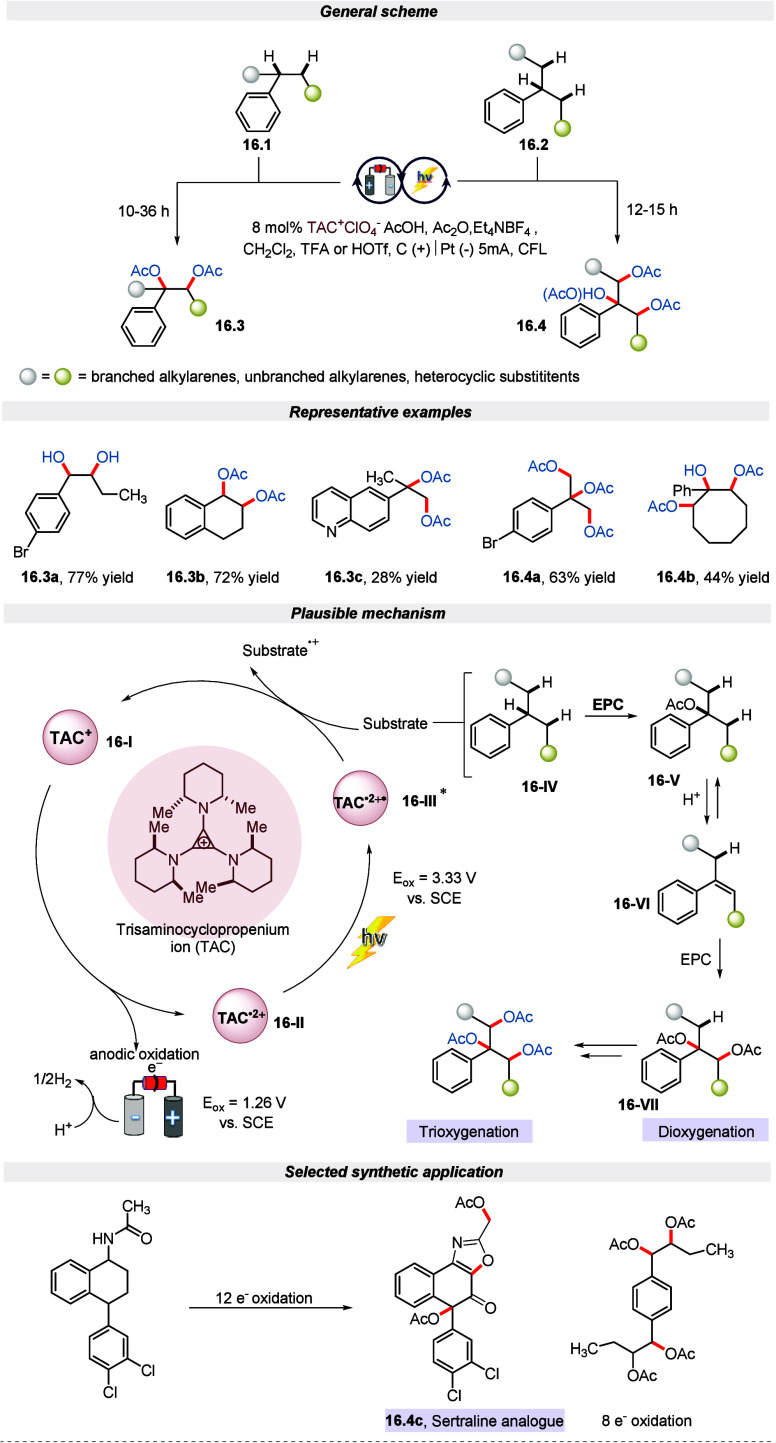
Electro-photochemical Oxygenation of Multiple C(sp^3^)–H Bonds

In 2023, Lambert et al. exploited an electro-photocatalytic approach catalyzed by a TAC cation for the oxygenation of multiple adjacent C–H bonds to their corresponding di- or triacetoxylates using inexpensive acetic acid as the oxygen source.^77^ The author noted that the use of the stronger HOTf acid in the case of the branched substrate with multiple C–H bonds leads to a third C–H oxygenation and results in a new trioxygenated product. The reaction successfully engaged a diverse range of branched and unbranched benzylic substrates containing a variety of functional groups and offered moderate to good yield. As illustrated in Scheme 16, the excited radical dication **16-III** is a powerful oxidant that can oxidize the substrate to a radical cation to undergo further reactions. The substrate **16-IV** could be converted to monooxygenated intermediate **16-V** under acidic electrophotocatalysis (EPC conditions). This intermediate **16-V**, in the presence of acid, undergoes elimination to generate an olefin **16-VI**, which encounters a second EPC oxidation to furnish deoxygenated adduct **16-VII**. Furthermore, this approach of peroxygenation has proven to be useful in creating biologically relevant structures such as analogs of Sertraline. Additionally, the author has demonstrated the scalability of this method for reactions.

### 2.3 Functionalization of C(sp^3^)–H Bond into C(sp^3^)–N Bond

**Scheme 17:**
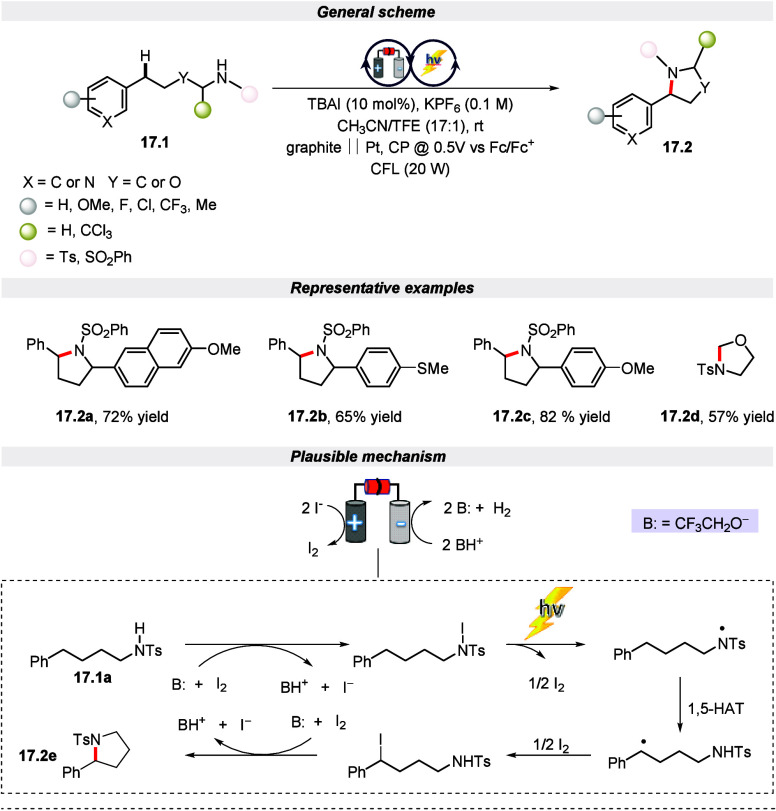
Electro-photochemical Amination of C(sp^3^)–H Bonds (Hofmann–Löffler–Freytag-Type Amination)

The formation of C–N bonds is important in synthetic organic chemistry because of its ubiquity in natural products, material science, and pharmaceuticals.^78−80^ Therefore, establishing strategies for the selective formation of C–N bonds via C–H activation is an appealing interest of synthetic communities.^81,82^ A notable instance of this type of reactivity is the Hofmann–Löffler–Freytag (HLF)^83^ reaction with manifold strategies such as electrochemical,^84,85^ photochemical,^86,87^ and others.^88−90^ In the related study, Stahl and co-workers achieved selective C(sp^3^)–H bond activation of *N*-alkyl sulphonamide and imidazole-based substrates for the formation of pyrrolidine and oxazoline derivatives via the HAT mechanism (Scheme 17).^91^ This Hofmann–Löffler–Freytag-type amination was conducted in an undivided cell with CFL illumination using tetrabutylammonium iodide (TBAI) as the mediator. The reaction is initiated by the anodic oxidation of I^–^ to I_2_ to form dihydrogen and a Brønsted base. This base promotes iodination of the N–H substrate, which further leads to a photoactive intermediate that undergoes homolytic cleavage of iodine to give an N-centered radical upon irradiation. Following this, a benzylic radical is formed after the HAT process that traps iodine to generate an alkyl iodide intermediate. This intermediate further undergoes base-promoted nucleophilic attack by nitrogen to deliver the final product. Hence, the electro-photochemical pathway could generate more powerful oxidants at lower electrode potentials, allowing for a C–H/N–H dehydrogenative coupling reaction with a wide functional group tolerance.

**Scheme 18:**
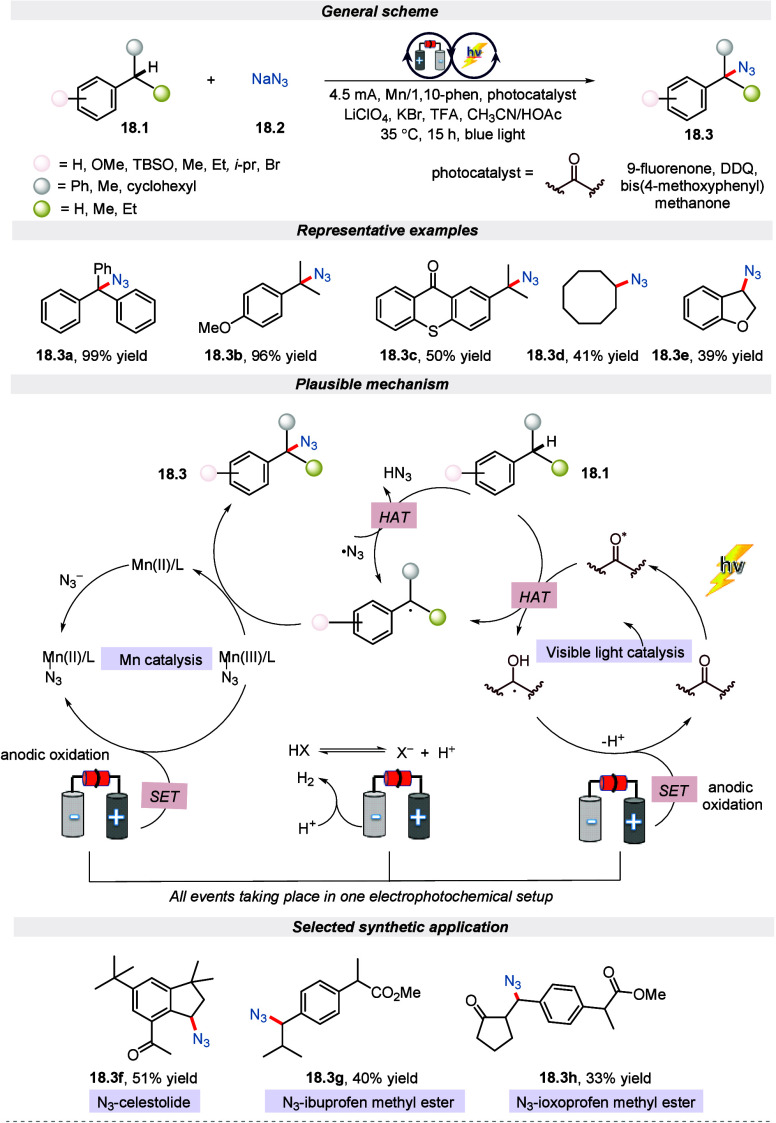
Electro-photochemical Azidation of Benzylic C(sp^3^)–H Bonds

Organic molecules that contain azides are incredibly versatile and essential compounds with numerous applications in chemical biology, material science, and pharmaceutical research.^92−95^ As a result, synthesizing this class of molecules is considered crucial for synthetic chemists.^96^ Considering its wide applications, Lei and the group reported an oxidant-free approach for the azidation of substrates containing the C(sp^3^)–H bond by merging the electrochemical/photochemical technique.^97^ The elegant use of manganese catalysts along with NaN_3_ as an azide source in the electro-photochemical conditions has been demonstrated in this work. Gratifyingly, this approach was compatible with a diverse range of secondary/tertiary benzylic, aliphatic, and drug-molecule-based C(sp^3^)–H bonds-bearing molecules. It is clear from the proposed mechanism in Scheme 18 that the C(sp^3^) radical is formed by activating the C(sp^3^)–H bond through the HAT event on blue LED irradiation. On the other hand, the event taking place on the anodic surface involves the formation of an azide radical, oxidation of Mn(II)/L-N_3_ to Mn(III)/L-N_3,_ and regeneration of photocatalyst. Finally, azide transfer from complex Mn(III)/L-N_3_ to alkyl radical was achieved to deliver the azide product. Furthermore, to test the synthetic utility of this approach, the author performed azidation to synthesize N_3_-celestolide, N_3_-ibuprofen methyl ester, N_3_-ioxoprofen methyl ester, and many more. Moreover, it is important to note that this reaction could be implemented for larger-scale synthetic applications, as well.

**Scheme 19:**
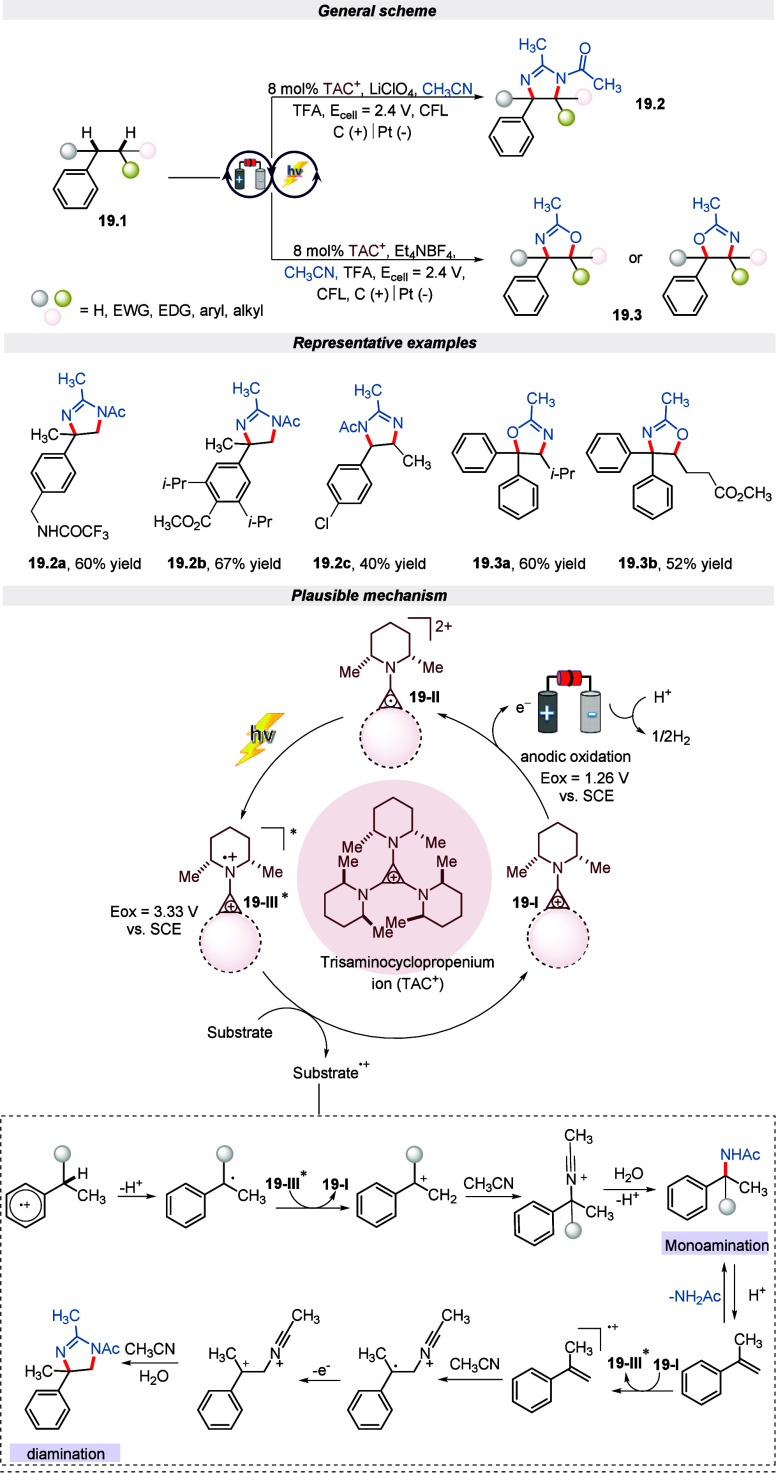
Electro-photochemical Deamination of Vicinal C(sp^3^)–H Bonds (Ritter-Type Amination)

Later in 2021, Lambert and co-workers reported an elegant example for the synthesis of biologically significant C–N containing complexes via C(sp^3^)–H bond activation. The reaction was accomplished by the Ritter-type deamination reaction of an α-branched substrate having vicinal C–H bonds in an electro-photocatalytic manner.^98^ The reaction begins with the anodic oxidation of TAC (I), which produces stable radical dication **19-II**. This radical dication absorbs light, resulting in an excited species **19-III** in an acidic environment. Species **19-III** then oxidizes the substrate through a single electron transfer, creating a radical cation. The radical cation undergoes deprotonation, followed by a second oxidation and solvolysis, to form the Ritter product. This product further undergoes reversible acid-catalyzed elimination to produce an α-methylstyrene derivative as showcased in Scheme 19. Finally, the dihydroimidazole or oxazoline product could be obtained by the single electron oxidation of α -methylstyrene with subsequent solvent trapping and oxidation events in Et_4_NBF_4_ and LiClO_4_ electrolytes, respectively. Additionally, it was noted that the deamination was compatible with a wide range of functionalities and practical utility for pharmaceutical synthesis, which was named Y5-receptor antagonists.

**Scheme 20:**
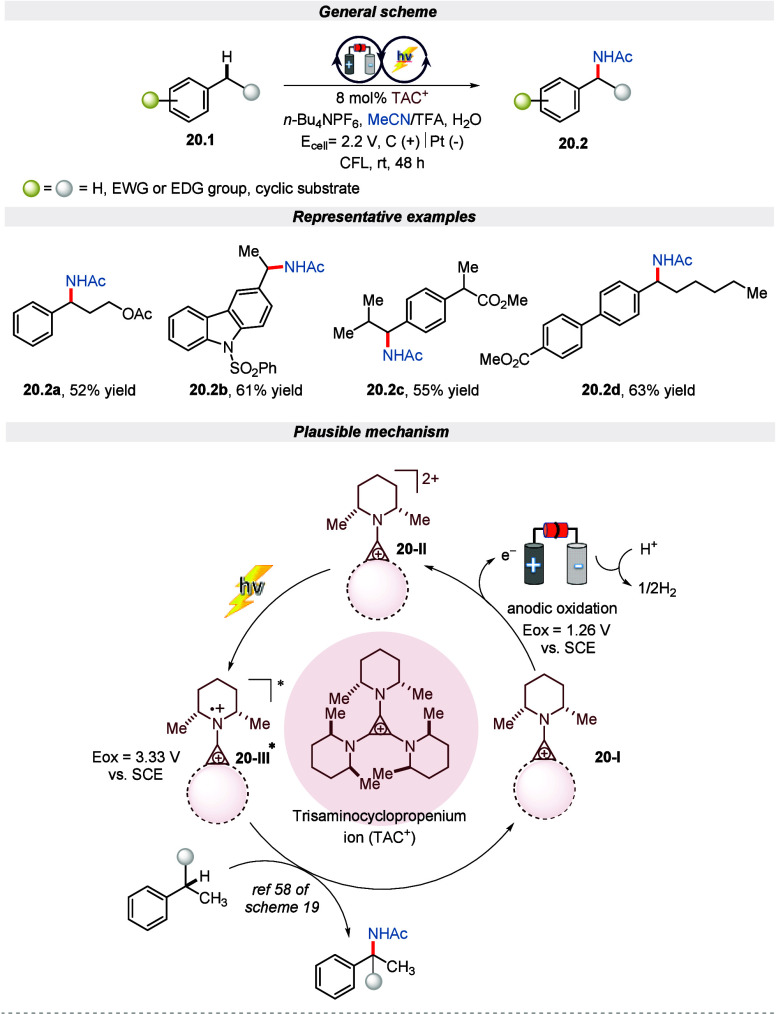
Electro-photochemical Monamination of Benzylic C(sp^3^)–H Bonds (Ritter-Type Amination)

In the same year, Lambert and his colleagues represented another Ritter-type monoamination of the unbranched substrate containing benzylic C (sp^3^)–H bonds under modified electro-photocatalytic conditions.^99^ The reaction proceeded well in TAC as a catalyst and *n*-Bu_4_NPF_6_ as an electrolyte in TFA/CH_3_CN as a solvent in divided cells under the irradiation of visible light. The protocol was tolerant with a wide range of functionalities in moderate to good yield. Furthermore, the author highlighted the contrasting facts for the occurrence of monoamination and deamination reactions. It was observed that during the deamination reaction, the benzylic acetamides generated initially undergoes an E1 elimination reaction to produce styrene, which then undergoes further oxidation and Ritter-type events to produce the dihydroimidazole product. Hence, in the case of branched benzylic substrates, the monoamination product is not observed because TFA leads to an effective elimination reaction. On the contrary, in the case of unbranched substrates, stronger acids are required for elimination, hence leading to a monoaminated product only as detailed in the mechanism (Scheme 20). Moreover, the gram-scale reaction furnishes the product in moderate yield by extending the reaction time.

**Scheme 21:**
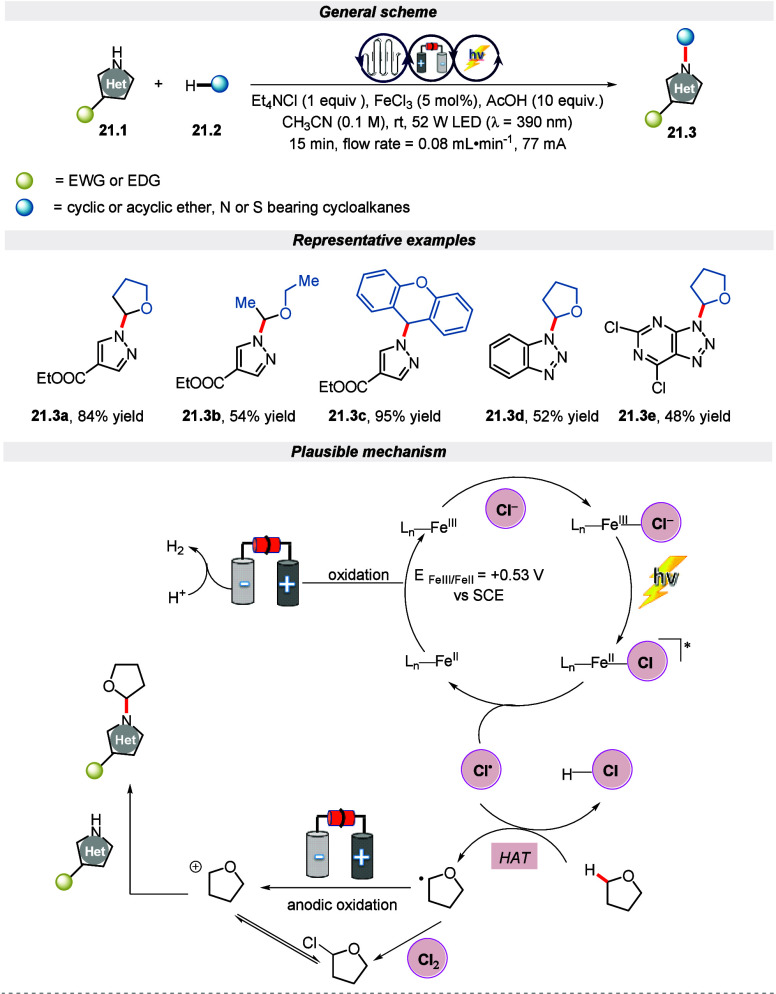
Electro-photochemical Heteroarylation of C(sp^3^)–H Bonds Utilizing Flow Chemistry

The EPRC techniques sometimes require lengthy reaction times, lowering the efficiency for scaling efforts. In this regard, flow electro-photochemistry (f-EPC) has emerged as an efficacious tool for an effective scale-up process while exposing the reaction mixture to more uniform radiation and better mixing without side reactions.^100,101^ Additionally, the flow reactors improve the reaction’s productivity and safety. Taking advantage of the flow chemistry, Noel et al. exploited a new synthetic approach for the heteroarylation of the C(sp^3^)–H bond via f-EPC that led to the functionalization of many organic complexes.^102^ The reaction proceeded under mild conditions employing a purple LED (λ = 390 nm) and in a galvanostatic mode (77 mA, 3.2 mA·cm^–2^, 6 F·mol^–2^) with FeCl_3_ as a photocatalyst in the presence of acid. It is worth mentioning that a trace amount of product was observed in the absence of either light or electricity. Further, the mechanism underlined in Scheme 21 suggested that the transformation occurred through integration of HAT via LMCT photocatalysis and electrochemical oxidation. The chlorinated iron complex gets excited upon irradiation with purple light, followed by the generation of chlorine radicals and reduced iron species that are further oxidized to Fe(III) species to close the cycle. Simultaneously, the chlorine radical transforms the substrate containing the C(sp^3^)–H bond to an α-oxy alkyl radical bearing substrate via the HAT process. Further, the so formed α-oxyalkyl radical is oxidized to cation, which is prone to nucleophilic attack by the *N*-heteroatom to form the desired product. Remarkably, the integration of flow chemistry leads to efficient scalability with a decent yield.

### 2.4 Functionalization of C(sp^3^)–H Bond into C(sp^3^)–B/P Bond

**Scheme 22:**
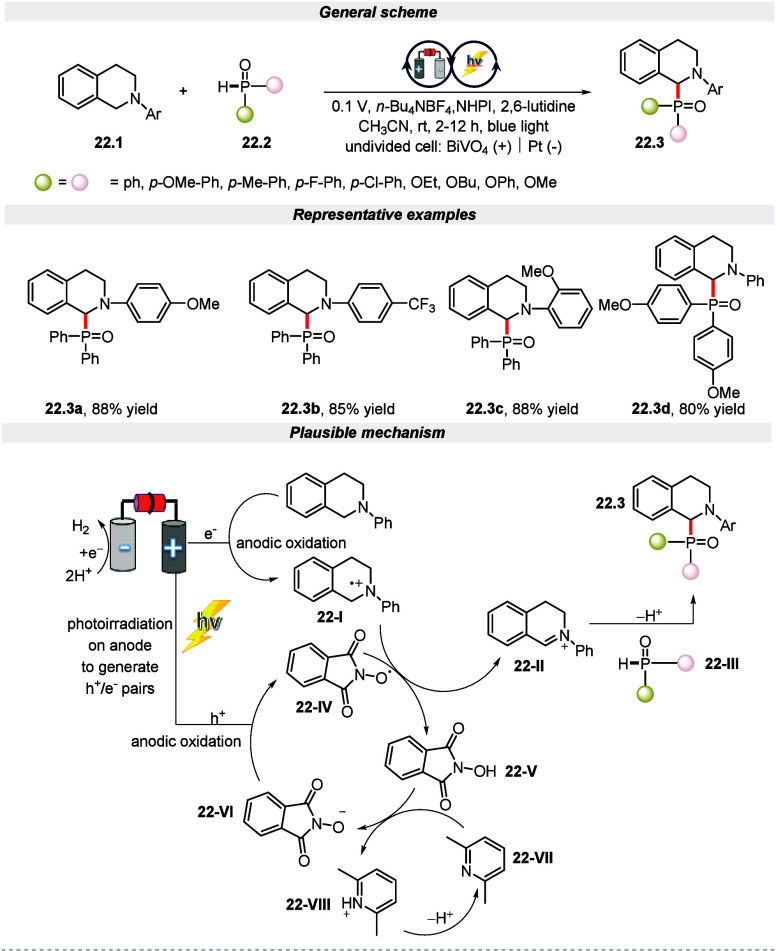
Electro-photochemical Phosphorylation of C(sp^3^)–H Bonds

Photoredox and electrochemistry have been demonstrated for the construction of C(sp^3^)–P bond through phosphorylation reactions.^103,104^ In 2019 for the first time, Wu and co-workers unlocked a new mechanistic pathway that enables the formation of C–P bonds by employing EPRC methodology.^105^ Using a Pt plate as the counter electrode and BiVO_4_ as the working electrode with a cell potential of 0.5 V (vs Ag/AgCl), the phosphorylation reaction via activation of the C(sp^3^)–H bond was conducted. It was found that *N*-hydroxyphthalimide (NHPI) is crucial for this process. It reduces the potential required to generate the photocurrent of 5 mA and increases the yield of the product. The mechanism portrayed in Scheme 22 indicates the generation of hole–electron pairs by the irradiated BiVO_4_ anode. This hole on the anodic surface oxidizes the substrate to radical cation intermediate **22-I** and phthalimide-*N*-oxyl (PINO) radical **22-IV**. The PINO radical **22-IV** is regenerated to NHPI **22-V** by extracting a hydrogen atom from the ammonium radical cation **22-I**, which is converted to an iminium ion **22-II**. Finally, this iminium ion is prone to nucleophilic attack by diphenylphosphine oxide to deliver the required product. Additionally, it is worth mentioning that this strategy for the formation of the P–C bond is amenable to a good range of functional groups with a wide substrate scope.

**Scheme 23:**
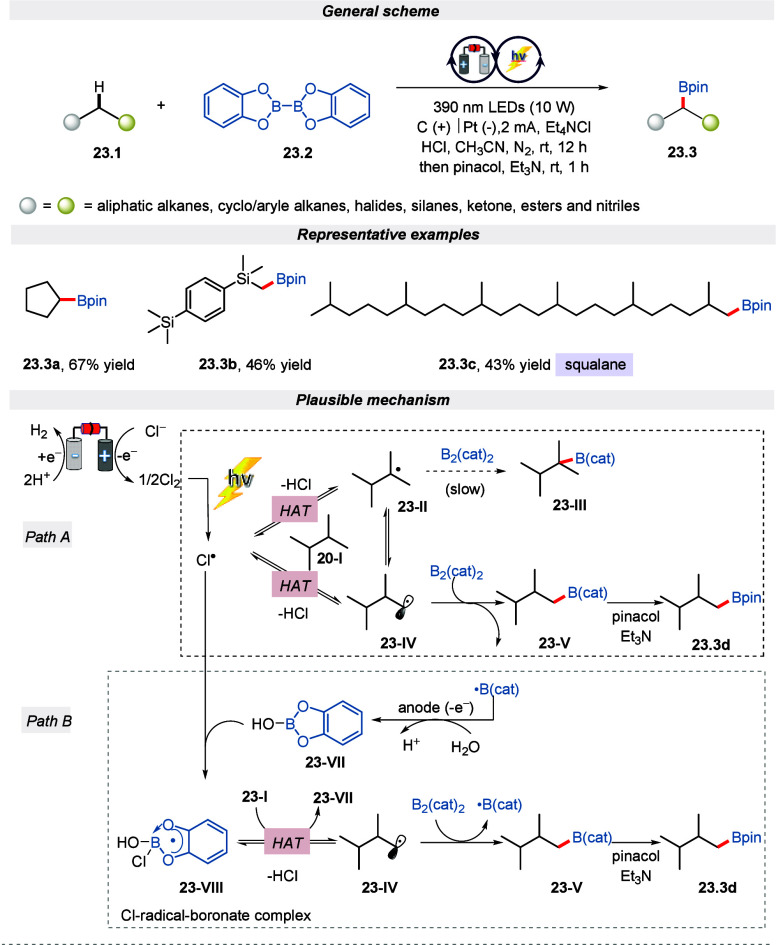
Electro-photochemical Borylation of C(sp^3^)–H Bonds

Alkyl/aryl boron compounds are the cornerstones of synthetic organic transformations along with applications in pharmaceuticals and agrochemicals for decades.^106^ Due to their significance, extensive research has been carried out on their synthesis via C(sp^3^)–H borylation. However, most of the current methods rely on precious metal catalysts or external oxidants to cleave C(sp^3^)–H bonds. Nonetheless, researchers are constantly exploring new approaches, which could be more efficient and cost-effective soon.^107−110^ In view of this, Xia and his group reported an oxidative electro-photochemical protocol for synthesizing aliphatic boronate ester via C(sp^3^)–H borylation (Scheme 23).^111^ The reaction involves tetraethylammonium chloride as electrolyte and a chlorine source with HCl as a proton source in an undivided cell under the irradiation of 390 nm LEDs. Noticeably, this synthetic protocol could tolerate a wide range of hydrocarbons, such as halides, silanes, ketones, esters, and nitriles. Furthermore, it was underlined that steric hindrance plays a vital role in the regioselectivity of the reaction. The reaction selectively occurs at the distal methyl position according to Aggarwal’s finding.^112^ To realize whether the radical intermediate is generated via the intramolecular HAT process, an EPR experiment was performed, which confirmed the result. Additionally, the mechanism outlined in Scheme 23 depicts two pathways for the occurrence of the reaction. Initially, the Cl^–^ is oxidized to Cl_2_ which, upon irradiation, is converted to chlorine. This chlorine radical then reacts with the substrate through HAT, forming unhindered radical **23-IV**. The radical **23-IV** then reacts with bis(catecholato)diboron (B_2_(cat)_2_), resulting in the formation of boronate ester **23-V** and a ligated boryl radical. Similarly, in path B the so-formed Cl-radical-boronate complex **23-VIII** undergoes the HAT process to form boronate ester **23-V**. Finally, the boronate ester formed via paths A and B reacts with pinacole and a base to give the desired product.

**Scheme 24:**
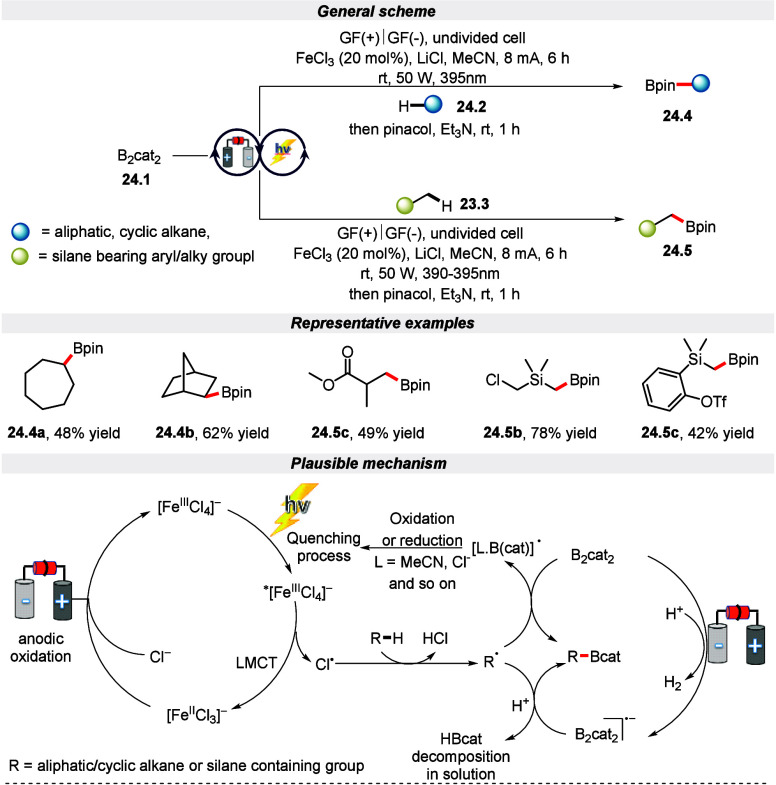
Electro-photochemical Borylation of C(sp^3^)–H Bonds

Recently, Lu and his colleagues demonstrated a method for achieving borylated compounds via C(sp^3^)–H borylation of alkanes using an electrophotocatalytic strategy (Scheme 24).^113^ The standout aspect of this transformation is that an ultralow oxidation potential is applied to activate inert alkanes to enable C(sp^3^)–H borylation under very mild conditions. The protocol allows for the synthesis of a wide range of alkyl or α-silyl boronic esters, demonstrating good tolerance for various functional groups, including cyclic, aliphatic, and benzylic groups. The process outlined in Scheme 24 indicates that the catalyst FeCl_3_ initially forms a coordination complex with a chloride anion in the solution, leading to the formation of [FeCl_4_]^−^. Subsequently, this species generates a highly reactive chloride radical through the process of LMCT when it is exposed to purple LED light. The chloride radical efficiently undergoes hydrogen abstraction by the alkane, resulting in the formation of a highly reactive alkyl radical. Simultaneously, the reduction of B_2_cat_2_ at the cathode results in the generation of the B_2_cat_2_ radical anion. This species subsequently engages in a coupling reaction with the reactive alkyl radical, leading to the formation of the targeted C(sp^3^)–H borylation product.

### 2.5 Functionalization of C(sp^3^)–H Bond to sp^2^ Carbon (Carboxylic and Carbonyl Group)

**Scheme 25:**
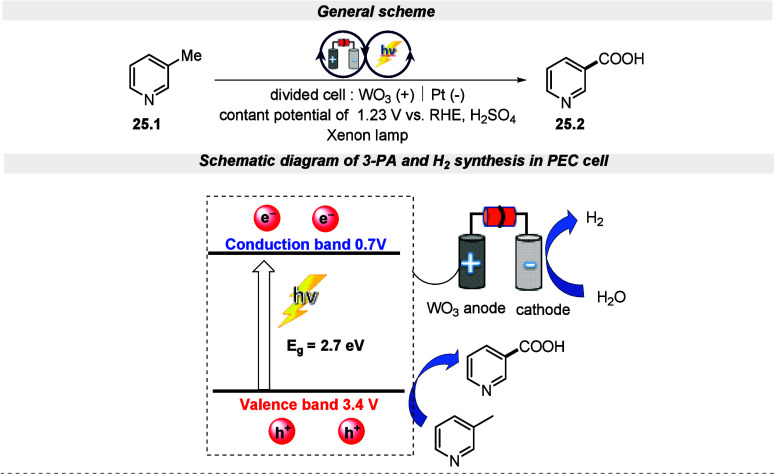
Electro-photochemical Synthesis of Aryl Carboxylic Acid via Oxidation of C(sp^3^)–H Bonds

In 2019, Zhou and fellow researchers introduced a highly effective EPRC method for synthesizing 3-pyridine-carboxylic acid while simultaneously producing hydrogen through a WO_3_ film photoanode-based dual chamber PEC cell.^114^ It is worth highlighting that the oxidation of 3-methylpyridine to 3-pyridinecarboxylic acid is co-initiated by the H_2_O_2_ intermediate and photogenerated holes that are generated on the WO_3_ film photoanode by the two-hole pathway of water oxidation. Furthermore, it was highlighted that the Cr_2_O_7_^2–^/Cr^3+^ redox pair also helps to efficiently mediate and oxidize the 3-methylpyridine to 3-pyridine-carboxylic acid (Scheme 25).

**Scheme 26:**
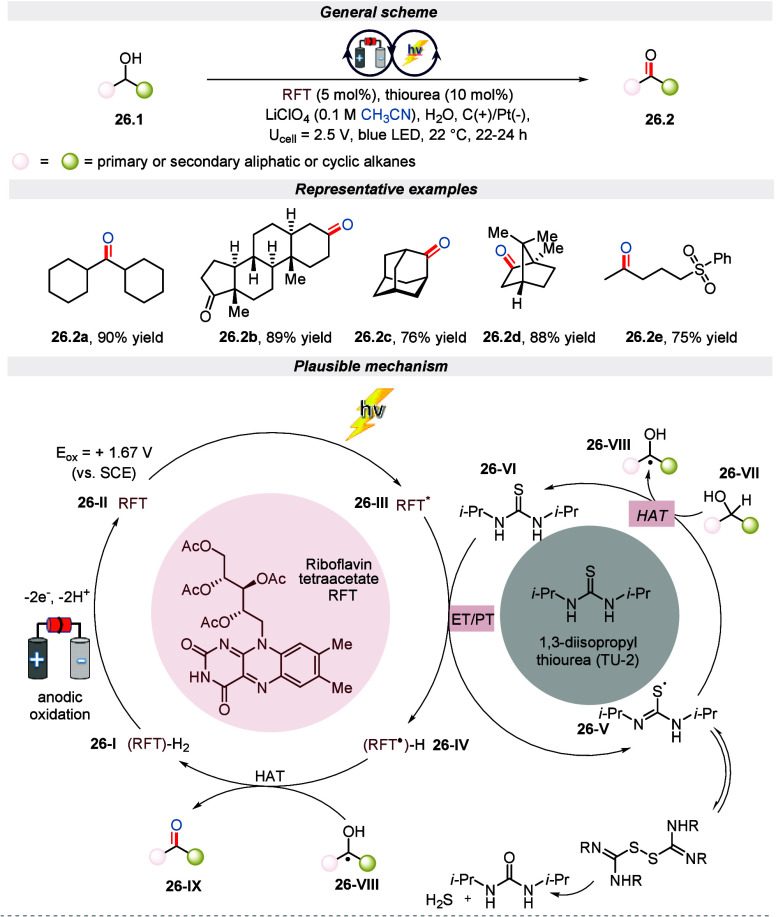
Electro-photochemical Oxidation of Aliphatic or Cyclic Alcohols Bearing C(sp^3^)–H Bonds

In the same year, Lin and colleagues used an electro-photochemical process to oxidize primary and secondary aliphatic or cyclic alcohols in a novel approach to C(sp^3^)–H bond functionalization.^115^ This method utilizes riboflavin-based photocatalysts and thiourea as cocatalyst in an undivided electrochemical cell, which is exposed to blue light. It has been successfully applied to a diverse range of primary and secondary alcohols, demonstrating the broad applicability of this protocol. Through a series of control experiments, the authors discovered that the absence of either light, electricity, thiourea, or riboflavin tetraacetate (RFT) would halt the reaction. Further, Scheme 26 shows that when irradiated, an electron transfer and proton transfer reaction occurs between excited RFT* and 1,3-diisopropyl thiourea (TU-2), producing thiyl radical **26-V** and semiquinone form (RFT^•^)-H. This thiyl radical then captures hydrogen atoms from the substrate **26-VII** to form the corresponding radical **26-VIII**. The radical **26-VIII** reacts with (RFT^•^)-H and ultimately produces the desired product.

## 3 Conclusion and Perspective

C(sp^3^)–H bond activation by electrophotochemistry uses electrons and photons as sustainable redox agents and opens new mechanistic routes for direct formation of target molecules which significantly shortens synthetic routes. The approach often exhibits good selectivity and accomplishes transformations that were previously not possible. Despite remarkable recent progress, the electrophotochemical activation of C(sp^3^)–H bonds is still in its initial phase. Here we outline the current limitations of electrophotocatalyzed C–H bond activation and suggest future research directions. (i) The current established strategies for incorporating functional groups, such as trifluoromethyl, halogens, methoxy, amino, hydroxy, nitro, methylamino, ethoxy, and carbonyl, which are highly relevant in medicinal chemistry via electrophotocatalyzed C(sp^3^)–H bond activation are limited. Thus, there is a need for further exploration to increase the scope of substrate classes and their widespread application in late-stage functionalization. (ii) The enantioselective or asymmetric synthesis by electrophotocatalytic C(sp^3^)–H bond activation has encountered limited success thus far. Notwithstanding, further research is necessary to address the challenges inherent in this method. (iii) Electrode materials and photocatalysts greatly influence electrophotochemical reactions. As such, there exists a pressing need to develop and design improved electrodes and photocatalysts that offer better stability and catalytic reactivity. Such advancements will effectively expand the domain of drug discovery through the electro-photocatalytic C(sp^3^)–H bond activation approach. (iv) Many researchers agree that although EPRC techniques are useful, they can sometimes be time-consuming and create obstacles for scaling efforts. In this regard, flow electro-photochemistry (f-EPC) has emerged as a better alternative for an effective scale-up process. By using f-EPC, the reaction mixture is exposed to more uniform radiation and better mixing without side reactions, leading to improved productivity and safety of the reaction. However, f-EPC-based C(sp^3^)–H bond activation reports are only a few. (v) Thus, far, most of the chemical reactions can only be executed on a relatively small scale owing to the limitations of reaction efficiency and reaction setup. We envisage that inert bond transformations, such as C(sp^3^)–H bonds, will be crucial for large-scale and industrial production of chemicals in the future. Overcoming these challenges would undoubtedly lead to advancements in chemical synthesis, improving overall efficiency, and reducing the cost of chemical production. Additionally, we would like to highlight that Reactions in which photochemical and electrochemical steps are combined and operate synergistically are mechanistically very complex. Investigations elucidating the effects of the various parameters must be therefore done with great care to avoid incorrect conclusions, difficulties in reproducibility, or incomplete mechanistic pictures. Control experiments should always include the omission of either irradiation or the electrochemical bias, but also the effect of variation of irradiation wavelength and intensity, and changes in the electrochemical parameters including electrode materials should be carefully evaluated. A detailed description of the experimental setup and parameters is essential to allow others to reproduce the results. Proposals of reaction mechanisms should always be considered as a scenario that describes all experimental observations correctly, but alternative pathways should be mentioned, if they cannot be excluded.

Hence, this comprehensive review provides an insightful analysis of the diverse range of synthetic methods available for electrophotocatalyzed C(sp^3^)–H bond activation chemistry. The review not only highlights the innovative and practical applications of this field but also urges researchers to further explore and investigate its potential in various domains. The study aims to motivate the synthetic community to leverage electrophotocatalytic C(sp^3^)–H bond activation chemistry to develop sustainable strategies for functionalization reactions and complex organic molecule synthesis.

## ABBREVIATIONS

PRC: Photoredox catalysis
EC: Electrocatalysis
HAT: Hydrogen Atom Transfer
EPRC: Electro-Photo Redox Catalysis
BDE: Bond dissociation energy
TAC: trisaminocyclopropenium ion
TBADT: tetrabutylammonium decatungstate
AQDS: disodium anthraquinone-2,7-disulfonate
NFSI: *N*-fluoro-succinimide
AQ: anthraquinone
LMCT: ligand-to-metal charge transfer
SET: single electron transfer
NCS: *N*-chloro succinimide
RVC: Reticulated vitreous carbon
TBAI: tetrabutylammonium iodide
HLF: Hofmann–Löffler–Freytag
NHPI: *N*-hydroxyphthalimide
RFT: Riboflavin tetraacetate
f-EPC: flow electro-photochemistry
